# Constitutively active androgen receptor supports the metastatic phenotype of endocrine-resistant hormone receptor-positive breast cancer

**DOI:** 10.1186/s12964-020-00649-z

**Published:** 2020-09-18

**Authors:** Shaymaa Bahnassy, Hariprasad Thangavel, Maram Quttina, Ashfia Fatima Khan, Dhanya Dhanyalayam, Joan Ritho, Samaneh Karami, Jing Ren, Tasneem Bawa-Khalfe

**Affiliations:** 1 Center for Nuclear Receptors & Cell Signaling, Department of Biology & Biochemistry, University of Houston, 3517 Cullen Blvd, SERC Bldg, Rm 3010, Houston, TX 77204-5056 USA; 2 Department of Pharmacy Practice and Translational Research, College of Pharmacy, University of Houston, Houston, TX 77204 USA; 3 Department of Biology, Stanford University, Stanford, CA 94305 USA; 4 Center for Precision Medicine, Department of Medicine, University of Missouri, Columbia, MO 65212 USA

**Keywords:** HR+ breast cancer, Endocrine resistance, Androgen receptor, SUMO, Enzalutamide

## Abstract

**Background:**

Hormone receptor positive (HR+) breast cancer (BCa) is the most frequently diagnosed subtype. Acquired and intrinsic resistance to conventional endocrine therapy (ET) commonly occurs and prompts incurable metastatic disease. Hence, ET-resistant (ET-R) HR+ BCa presents a therapeutic challenge. Previous studies show elevated androgen receptor (AR) that supports resistance to ET tamoxifen and correlates with HR+ BCa metastasis. Yet surprisingly, studies with AR-blocker enzalutamide (Enz) in ET-R HR+ BCa present conflicting results. We now report that a constitutively active, unique from canonical Enz-targeted, AR accumulates in endocrine resistant HR+ BCa cells.

**Methods:**

AR protein profiles in acquired and intrinsic ET-R HR + -BCa were defined with cell-free modification tests, in-house in-vivo SUMOylation assays, and PLA imaging. Genomic activity of native AR and modified-AR mimetic was tested with reporter assays and limited transcriptome analysis. Spheroid growth and migration studies were used to evaluate inhibitory actions of Enz and combinatorial therapy.

**Results:**

Sustained higher molecular weight SUMO-modified AR (SUMO-AR) persists in acquired and intrinsic ET-R BCa cell lines. Concurrently, SUMO isoforms and global SUMO-modified proteome also accumulates in the same cell lines. We identified AR as a novel substrate for the SUMO-E3 ligase HSPB1/Hsp27. Independent of ligand, SUMO-AR is resilient to ubiquitin-mediated proteasomal degradation, enriched in the nucleus, readily chromatin-bound, and transcriptionally active. Constitutive SUMO-AR initiates a gene-expression profile that favors epithelial-mesenchymal transition. Enz combined with a SUMO inhibitor attenuates migration and metastatic phenotype of ET-R HR+ BCa.

**Conclusion:**

Targeting both unmodified and SUMO-modified AR prevents the metastatic progression of HR+ BCa with ET-R.

Video abstract

## Background

Endocrine therapy (ET) remains the first line cancer therapy for early-stage hormone-receptor positive (HR+) breast cancers (BCa). The selective estrogen receptor modulator (SERM), Tamoxifen (Tam) is effective for pre−/peri-menopausal women while post-menopausal women receive anti-estrogen aromatase inhibitors (AI). However, intrinsic or acquired ET-resistance is common and equates to rapid onset of malignant metastatic disease. Hence, HR+ BCa unresponsive to conventional ET poses a new challenge with no targeted therapy available to prevent cancer progression.

The androgen receptor (AR) is emerging as a good therapeutic target for other advanced BCa subtypes [1]. Canonical AR functions with ligand androgen binding, subsequently translocate form the cytosol to the nucleus, associates with androgen response elements (ARE) in gene enhancer and/or promoter regions, and transcriptionally regulates corresponding genes [2]. This ligand-driven transcription factor is widely expressed in primary and metastatic breast tumors and specifically, 75% of HR+ BCa tissue samples express AR [1, 3]. Higher AR expression in HR + -patients correlates with reduced clinical response to ET tamoxifen and worse prognosis with greater cancer recurrence [4]. Induction of AR initiates Tam-resistance (TamR) in HR+ BCa cell models and promotes tumor growth in mouse xenografts [5], highlighting a role for AR in ET-R and disease progression [4–6]. However, previous studies using a second-generation AR antagonist Enzalutamide (Enz) in ET-R HR+ BCa present conflicting results. Treatment of MCF7-TamR cells with Enz, reduced growth and colony formation in one study [6] but showed no effect in another study [7]. In addition, Enz failed to reduce tumor growth of the HR+ ET-R patient-derived xenograft (HCI-13) [8]. In clinical trials, positive response of HR+ BCa patients to Enz is dependent on prior ET history. While combinatorial Enz and AI therapy improved progression-free survival for women with advanced HR+ BCa without prior ET history, patients previously on ET did not respond to the same Enz-AI combination [9]. Yet, ongoing clinical trials continue evaluating Enz alone or in combination with other endocrine therapies (NCT02676986, NCT02953860 and NCT02955394). This raises the question: how is the AR different in HR+ BCa patients previously treated with ET?

The AR is subject to several post-translational modifications (PTM) including Small Ubiquitin Like Modifier (SUMO)-PTM [10, 11]. Protein SUMOylation is the reversible covalent binding of SUMO to a lysine residue within a specific consensus sequence ψKXE (ψ is a hydrophobic residue and X is any amino acid) [12]. Upon ligand activation, AR residues K386 and K520 are targets for the 3 SUMO paralogs SUMO-1, − 2, and − 3 [13, 14]. Also, SUMO enzymatic components like deSUMOylases and SUMO-E3 ligases dictate the level of SUMOylated AR. Previously we demonstrated that elevated deSUMOylase SENP1 prevents accumulation of SUMOylated AR in prostate cancer [15–17]. This is important as high-throughput AR functional studies show that SUMO-PTM regulates the chromatin occupancy and transcriptional activity of ligand-activated AR in a gene dependent manner [18].

We now report that high levels of SUMOylated AR persist independent of AR-ligand in ET-R HR+ BCa cells. In these BCa lines, SUMO-PTM supports constitutive AR genomic activity to promote metastatic transformation. Concurrent targeting of SUMOylated AR improves the inhibitory actions of Enz on the drug-resistant HR+ BCa.

## Methods

### Cell culture and transfections

Sensitive (MCF-7) and resistant (derived MCF-7-TamR or intrinsically resistant GI-101A and GILM2) human breast carcinoma cell lines to ET were cultured in Dulbecco’s modified Eagle’s medium (DMEM; Gibco) supplemented with 10% FBS and 1% penicillin/streptomycin at 37 °C in a humidified atmosphere containing 5% CO_2_. MCF-7-TamR cells were generated in our lab by chronically treating MCF-7 with 1 μM 4-OH-tamoxifen (Sigma-Aldrich) for over 6 months. After developing resistance, the derived cells were used and continuously cultivated in presence of tamoxifen. All transient transfections were performed with Lipofectamine 2000 (Invitrogen) according to manufacturer instructions.

### Immunoprecipitation (IP) and Chromatin-Immunoprecipitation (CB/IP) assays

After harvesting and lysing the cells in a buffer containing protease inhibitor (Roche) and *N*-ethylmaleimide (Sigma-Aldrich), proteins were either immunoprecipitated from whole-cells or chromatin fractions using the same protocol described before [19]. Immunoprecipitated proteins were then bound to Protein A-agarose beads (Millipore) and samples were resolved on SDS-PAGE gels and immunoblotted with specific antibodies.

### SDS-page and Western blot analysis

Whole-cell or immunoprecipitated protein extracts were denatured, resolved on SDS-PAGE gels and transferred onto PVDF membranes (BioRad) using a wet transfer apparatus. After blocking, blots were probed overnight and the following primary antibodies were used: AR (sc-816), Ubiquitin (sc-8017) and GAPDH (sc-8017) from Santa Cruz Biotechnology, AR441 (MA5–13426) and Hsp27 (MA3–015) from ThermoFisher, SUMO1 (49,305, Cell Signaling), SUMO2/3 (ab81371, Abcam), PIAS1 (49,305, Cell Signaling), and β-actin (Sigma-Aldrich). Antigen–antibody complexes were then detected by the chemiluminescence Western Lighting Plus-ECL reagent (PerkinElmer).

### Statistical analysis

All results are expressed as mean ± SEM. Prism 7 (GraphPad Software) was used for data analysis. Student’s two-tailed *t-test* or one-way ANOVA followed by Tukey’s multiple comparison test was employed to evaluate statistical significance between groups and *p*-values < 0.05 were considered statistically significant.

Remaining methods used in this study are found in Additional file 1: supplemental materials and methods.

## Results

### SUMO-modified AR persists in ET-R BCa

**Fig. 1:**
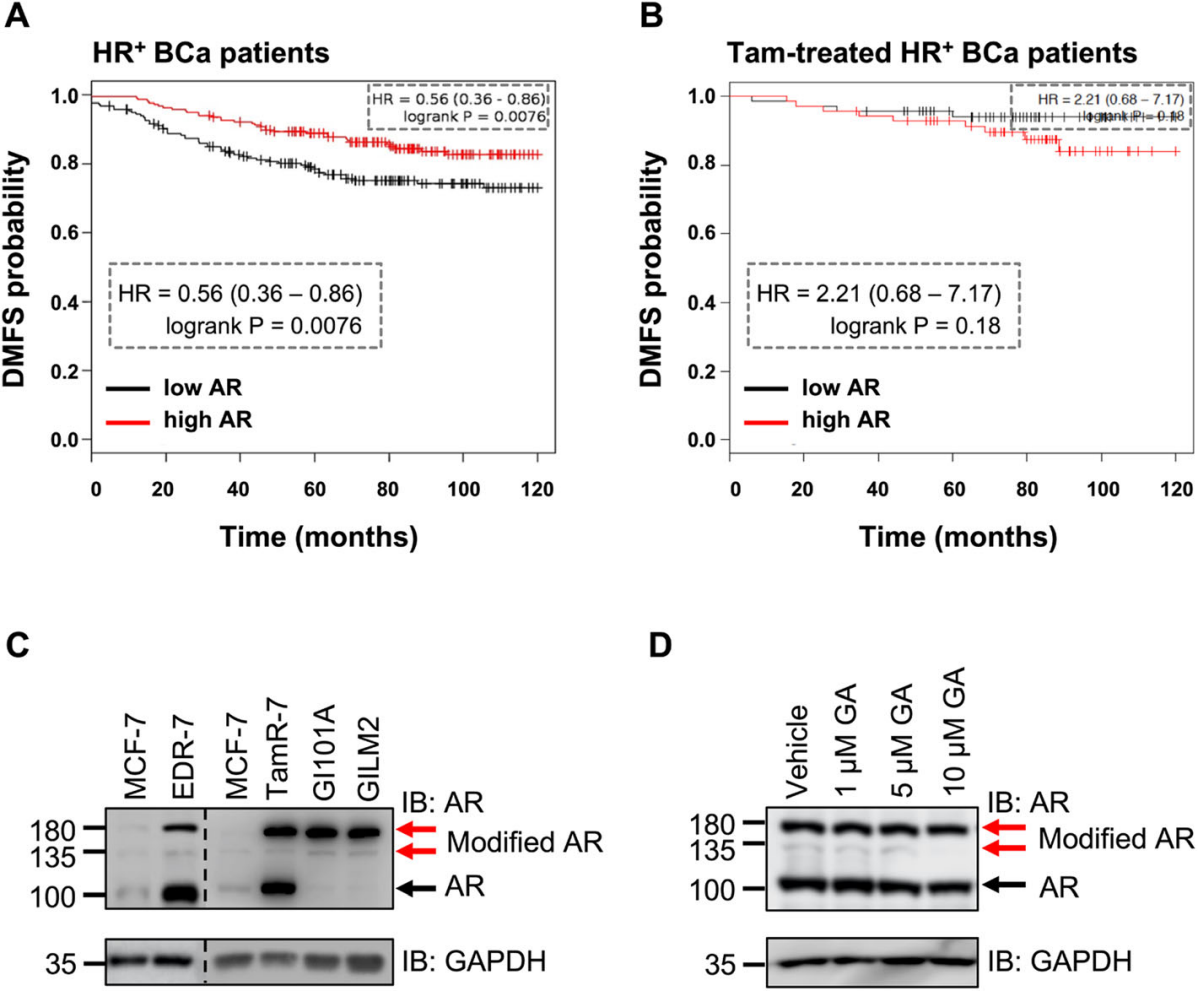
AR is highly modified in ET-R BCa. **a**-**b** High expression of AR correlates with better DMFS for HR^+^ BCa patients not receiving Tam therapy. Generated plots from KM Plotter database for BCa (www.kmplot.com) illustrate 10-year DMFS for: (**a**) HR + -BCa and (**b**) Tam-treated HR + -BCa patients. Plots separate high and low gene expressions. Both HR, representing hazard ratio at 95% confidence, and log-rank *P* values are enlarged in the dotted box. **c** Endogenous AR protein is highly modified in long-term estrogen deprived (EDR-7), acquired (TamR-7) and intrinsic TamR-BCa cell lines (GI-101A and GILM2). Blot represents three independent experiments; arrows indicate modified and unmodified AR. **d** Ginkgolic acid (GA) inhibits SUMO-modification of AR in a dose-dependent manner. TamR-7 cells were treated with increasing concentrations of GA and immunoblot represents two independent experiments

Elevated RNA levels of AR were previously reported in metastatic TamR BCa tumors [5]. We analyzed AR expression in publicly available survival datasets from the Kaplan-Meier Plotter database [20], and interestingly observed a reversed pattern in distant-metastasis free survival (DMFS) of total versus Tam-treated HR+ BCa patients (Fig. 1a-b). Specifically, patients with high AR levels exhibit a better prognosis with lower probability to develop metastasis; inversely patients treated with Tam have a worse prognosis with higher susceptibility for metastatic onset (log-rank *p* = 0.0076, HR = 0.56 versus *p = 0.18*, HR = 2.21, respectively Fig. 1a-b).

Consistent with a previous report [7], both long-term estrogen-deprived cells (EDR-7) that mimic AI resistant HR+ BCa, and acquired TamR-7 cells, generated with continuous culturing of MCF-7 cells with 1 μM 4-OH-tamoxifen, express higher protein levels of unmodified AR at ~ 100 kDa when compared with endocrine therapy-sensitive (ET-S) parental MCF-7 cells (Fig. 1c). In addition, we observe two slowly migrating higher molecular weight bands (modified AR) elevated in both EDR-7 and TamR-7 along with the unmodified AR. Intrinsic TamR-BCa cell lines, GI-101A and its highly metastatic variant GILM2 [21, 22], also express the modified but not unmodified AR (Fig. 1c). Previous AR SUMOylation studies demonstrate analogous higher molecular weight bands corresponding to the two major SUMO acceptor sites of AR at lysine residues 386 and 520 [13]. To test if the modified AR in TamR cells is SUMOylated, TamR-7 cells were treated with ginkgolic acid (GA) to block the activity of SUMO-E1 and inhibit protein SUMOylation [23]. Increasing GA treatment reduced high molecular weight AR (Fig. 1d).

### A hyperSUMO environment exists in TamR-BCa

**Fig. 2:**
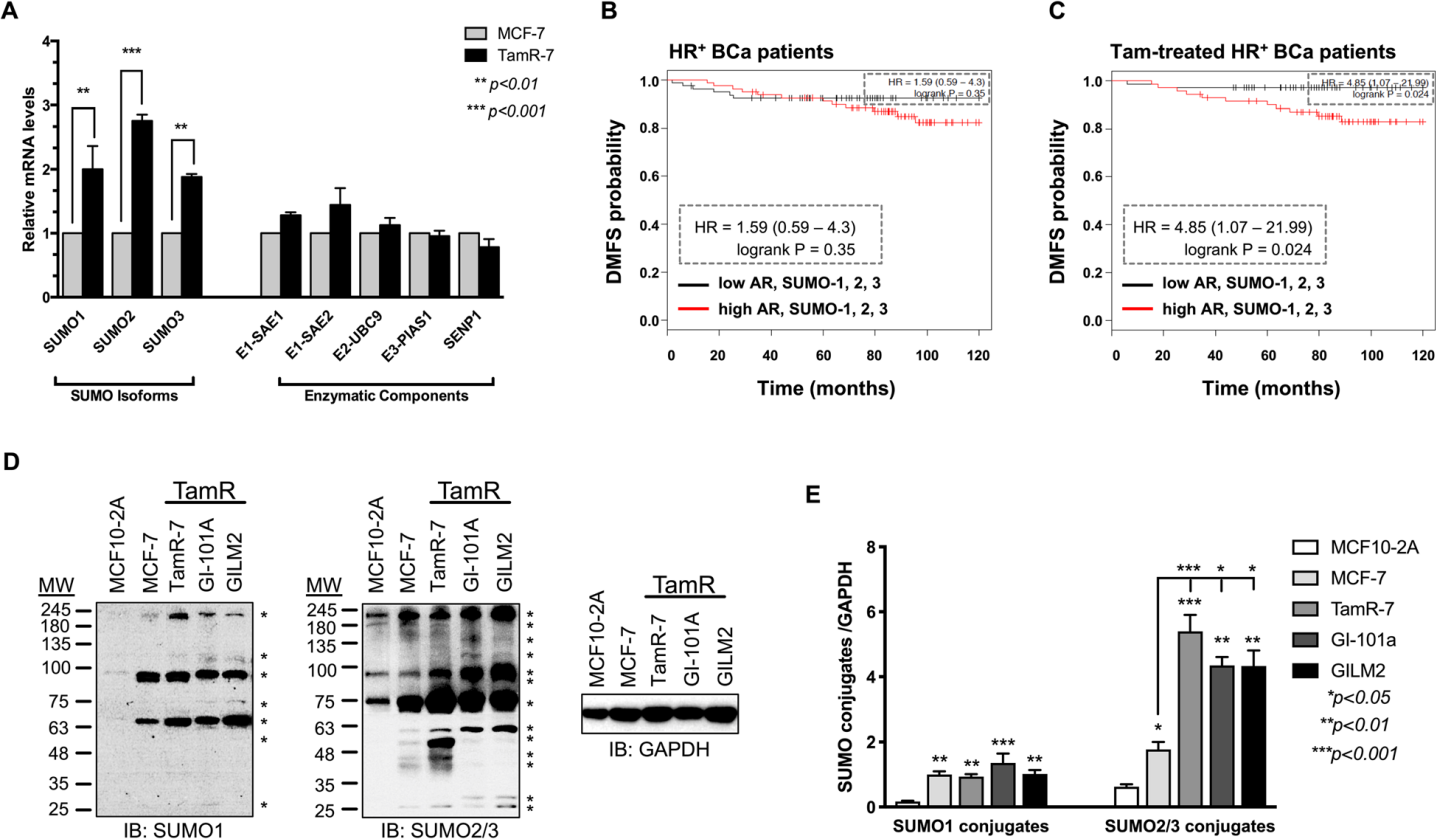
Global SUMOylation increases in TamR-BCa*.* **a** SUMO transcripts are significantly higher in TamR-7 BCa cells. Real-time PCR was performed using TamR-7 and parental MCF-7 cells. Graph represents fold-changes (2^ΔΔCt^ values) in gene expressions of 3 independent experiments and statistical significance in raw ΔCt values using Student’s t-test. **b**-**c** Tam-treated HR+ BCa patients expressing high levels of AR and SUMO isoforms show high probability for developing metastasis. Plots for: **b** HR + -BCa patients; (**c**) Tam-treated HR+ BCa patients illustrate 10-year DMFS. HR and log-rank P values are enlarged in dotted box. **d**-**e** Western blot analysis show enhanced global SUMO2/3 conjugates in acquired and intrinsic TamR-BCa cell lines; SUMO conjugates are marked by asterisks. **e** Blots from (D) were analyzed by densitometry using ImageJ from at least two independent experiments. Overall SUMO-1 or SUMO-2/3 conjugates were normalized to GAPDH and graphed as mean ± SEM. One-way ANOVA followed by Tukey’s multiple comparison test was performed to compare between groups and statistical significance is shown on the graph

We postulated that altered expression of SUMO paralogs and/or enzymatic components could support elevated levels of SUMOylated AR in drug resistant BCa cells. In TamR-7 versus TamS cells, transcript levels of all three SUMO isoforms significantly increased (Fig. 2a). In addition, we included components of the SUMO-enzymatic machinery that SUMOylate and/or interact with AR based on published literature and high-throughput screens [24–27]. SUMO-specific activating E1-SAE1/SAE2 dimers, conjugating E2 Ubc9, ligating E3 PIAS1, and deSUMOylase SENP1 enzymes are equivalently transcribed in TamS and TamR-7 cells (Fig. 2a). In contrast, the RNA levels for a canonical AR binding partner with proposed SUMO-E3 activity for other substrates Hsp27 is upregulated (*p < 0.05;* Additional file 1: Fig. S1A). To evaluate whether transcript changes correlate with disease progression, publicly available datasets were analyzed with KM plotter. Specifically, survival data shows that high SUMO levels directly correlate with high probability of metastasis in TamR-BCa patients (log-rank *p* = 0.027; Additional file 1: Fig. S1B). Moreover, Tam treated HR^+^-BCa patients with concurrent elevated levels of AR and SUMO exhibit a greater risk for developing metastasis (log-rank *p* = 0.024, HR = 4.85 in Tam-treated versus *p =* 0.35, HR = 1.59 for total HR^+^ patients, respectively Fig. 2c and b). Clearly ET treatment of HR+ BCa supports unique gene expression of the SUMO system.

Next, we determined if this induction of SUMO proteins equate to changes in the SUMO-modified proteome. As compared to noncancerous MCF10-2A cells, SUMO-1 and SUMO2/3 conjugated proteins are greatly enriched in all HR+ BCa lines (Fig. 2d and e). Interestingly, SUMO2/3 conjugates are favored in TamR HR+ BCa; specifically, acquired TamR-7 cells and intrinsic TamR GI-101A and GILM2 cells express greater SUMO2/3-conjugated proteomes than TamS MCF-7 cells (Fig. 2d-e). Hence, induction of SUMO isoforms supports the elevated SUMOylome in TamR-BCa.

### AR is a substrate for the SUMO E3 ligase Hsp27

**Fig. 3:**
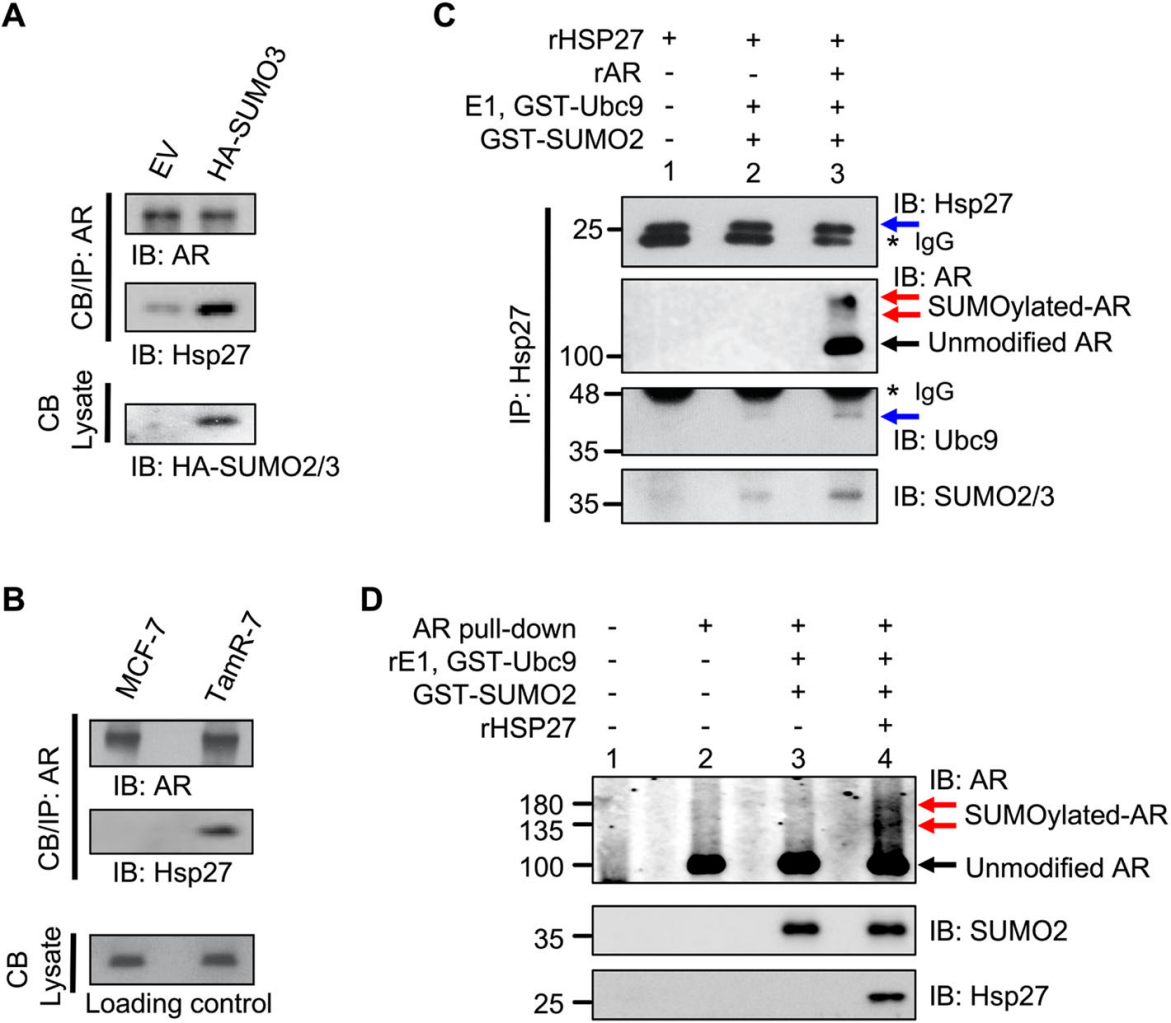
AR interacts with and is a substrate for the SUMO E3 ligase Hsp27. **a** Ectopic induction of a hyperSUMO environment in MCF-7 cells with HA-SUMO3 increases the interaction between AR and Hsp27. **b** AR-Hsp27 interaction complex is enhanced endogenously in the naturally occurring hyperSUMO environment of TamR-7 cells. **a**-**b** Interactions with chromatin-bound (CB) AR were analyzed by western blot. **c** Immunoprecipitation of Hsp27 was performed to assess its binding with AR, Ubc9 and SUMO-2/3 in a cell-free system. **d** SUMOylation of AR is enhanced under in vitro conditions when recombinant Hsp27 is added. Details are found in (Additional file 1)

We evaluated the relationship between AR and SUMO E3 ligases expressed in drug-resistant BCa cells; specifically, PIAS1, an established E3 [25, 28] versus Hsp27, a novel E3 for AR. Interestingly, Hsp27, but not PIAS1, transcript is elevated in TamR cells (Additional file 1: Fig. S1A and Fig. 2a) and this induction correlates with metastasis (log-rank *p* = 0.01 in Additional file 1: Fig. S1C and *p* = 0.82 in Fig. S1D). Also, induction of SUMO, analogous to native TamR conditions, enhances the interaction of AR with Hsp27, but not PIAS1 (Additional file 1: Fig. S2A). As SUMOylated AR in TamR-7 cells is chromatin bound (Additional file 1: Fig. S2B), co-immunoprecipitation studies were performed in chromatin fractions. We observe enhanced interaction of AR with Hsp27 in the chromatin fraction of SUMO-induced TamS MCF-7 cells (Fig. 3a) and natural hyperSUMO environments of TamR-BCa cells (Fig. 3b).

Next, we tested if Hsp27 can serve as a SUMO-E3-ligase for AR. As required for ligase activity, recombinant Hsp27 associates with the substrate AR, SUMO-E2 Ubc9, and SUMO2 protein (Fig. 3c). To test this further, we did in vitro SUMOylation assays of AR in presence and absence of Hsp27. Surprisingly, SUMO-2 by itself did not generate high-molecular-weight AR conjugates which were only observed when recombinant Hsp27 was added (lanes 3 versus 4, Fig. 3d). Hence, we show for the first time that Hsp27 potentiates AR SUMOylation via its SUMO E3 ligase activity.

### AR SUMOylation drives its chromatin binding

**Fig. 4:**
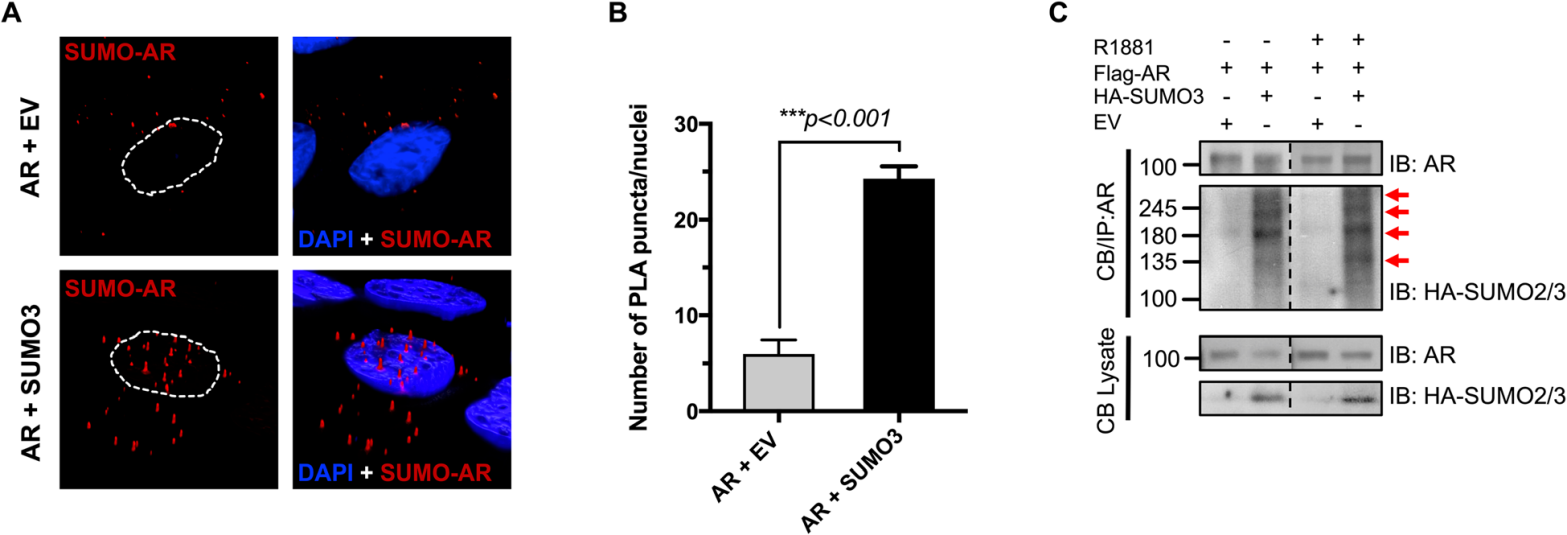
SUMOylation dictates the chromatin binding of AR. **a** PLA shows predominant nuclear localization of SUMOylated AR when a hyperSUMO environment is introduced. MCF-7 cells were co-transfected with AR + EV or SUMO-3. The DuoLink kit was used according to manufacturer’s instructions and total number of AR/SUMO complexes (red puncta) per nuclei were quantified using Image J. Represented images were derived from original images by deconvolution; white-dashed lines highlight the nuclei. **b** Graph represents quantification of nuclear red puncta as mean ± SEM. Student’s *t*-test was used to compare groups and ***p < 0.001 was considered statistically significant. **c** SUMO-3 enhances SUMOylation and recruitment of AR to chromatin even when cells were unstimulated with synthetic androgens (R1881). ET-R-like conditions were introduced in hormone-deprived MCF-7 cells by overexpressing AR with and without SUMO-3. After 24 h, cells were unstimulated or stimulated with 10 nM R1881. Chromatin-bound AR were immunoprecipitated, resolved by SDS-PAGE and immunoblotted with specific antibodies. SUMO-2/3 conjugates of AR are identified with red arrows

As SUMOylation is known to alter the subcellular localization of conjugated proteins, we performed proximity ligation assays to examine the SUMO-AR distribution. AR-SUMO conjugates (red puncta) is readily detected and shifts from the cytoplasm to the nucleus with the overexpression of SUMO3 in TamS MCF-7 cells (Fig. 4a). A significant 4-fold increase in the total number of AR/SUMO complexes per nuclei occurs under hyperSUMO conditions (*p <* 0.001; Fig. 4b). In vivo SUMOylation studies show that treatment with synthetic androgen R1881 enriches SUMOylated AR at the chromatin (Fig. 4c). However, and to our surprise, hyperSUMO environment in TamS cells also increase SUMOylated AR at the chromatin independent of androgen (Fig. 4c). We also observe endogenous SUMOylated AR in chromatin fractions in TamR cells (Additional file 1: Fig. S2B). Collectively, these results show that AR SUMOylation promotes chromatin enrichment independent of ligand activation.

Additionally, as inhibition of SUMOylation decreases modified AR protein levels (Fig. 1d), we investigated if and how the SUMO system stabilizes AR. First, cyclohexamide (CHX) pulse experiments were conducted to determine the rate of degradation of endogenous AR in TamR-7 versus TamS parental MCF-7 cells. Clearly, greater AR protein stability contributes to the greater population of AR in TamR-7; specifically, T_1/2_ > 24 h in TamR versus T_1/2_ ≈ 13 h in TamS (Additional file 1: Fig. S3A). Next, to test if SUMO supports AR stability, parental TamS MCF-7 were overexpressed with SUMO and then subjected to CHX studies. SUMO overexpression significantly reduces the rate of AR degradation (T_1/2_ ≈ 24 h) as compared to empty vector (T_1/2_ ≈ 8.2 h). However, SUMO overexpression produced no significant difference in the degradation rate of upper band modified AR (Additional file 1: Fig. S4B), suggesting that SUMO-modified AR is more resilient to degradation. Consistently, analogous SUMO condition also reduces AR ubiquitylation (Additional file 1: Fig. S4C). Hence, hyperSUMO conditions favors AR stability.

### AR SUMOylation drives androgen-independent genomic activity

**Fig. 5:**
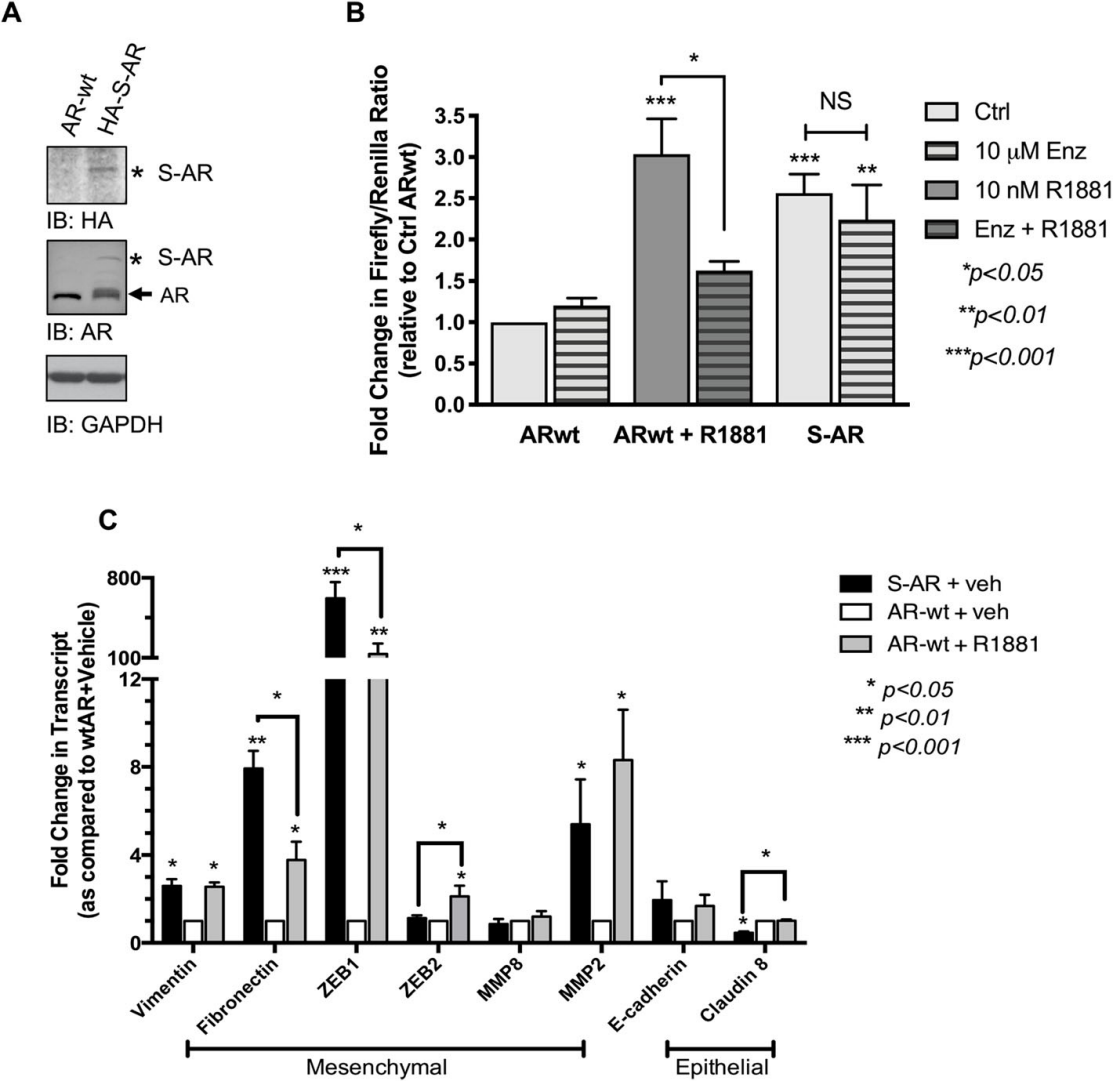
SUMOylation upregulates AR-dependent transcriptional activity and mesenchymal gene expression in BCa. **a** Characterizing the SUMO-mimetic AR construct by western blot; asterisk represents AR-SUMO conjugates. **b** MCF-7 cells were hormone-deprived for 48 h then transfected with probasin and renilla luciferase together with either ARwt or SUMO-fused AR (S-AR). The next day, cells were either untreated or treated with 10 μM Enz, 10 nM R1881 or a combination of both for additional 24 h then harvested and assayed for luciferase activity. Graph B represents fold changes in firefly/renilla ratios for at least three independent experiments, each performed in triplicates and asterisks denote significant changes using One-way ANOVA followed by Tukey’s multiple comparison test. **c** RNA from cells expressing ARwt or S-AR were used for real-time PCR to assess the expression of mesenchymal, epithelial markers and EMT inducers (ZEB1 and ZEB2). Graph represents fold-changes in indicated mRNA (2^ΔΔCt^ values) for 3 independent experiments; asterisks indicate statistically significant differences in raw ΔCt values when compared to the ARwt + vehicle group using Student’s *t*-test

Since SUMO enhanced the chromatin binding of AR (Fig. 4c), we postulated that AR SUMOylation likely impacts AR transcriptional activity. We generated a SUMO-mimetic AR (S-AR) to test if direct AR SUMOylation enhances AR transcriptional activity. Higher molecular weight bands of AR-SUMO conjugates are induced by S-AR but not ARwt (Fig. 5a). As observed previously in other cell lines [29], 10 nM of synthetic androgen R1881 stimulates ARwt transcription of a probasin-reporter vector in parental MCF-7 (Fig. 5b). Treatments with Enz significantly lower the R1881-mediated transactivation of ARwt. In absence of exogenous ligand, the S-AR mimetic exhibits substantially greater basal transcriptional activity than ARwt (*p < 0.001*; Fig. 5b), suggesting that addition of SUMO to AR directs AR transcriptional function. Enzalutamide treatments on the other hand, had no effect on the increased basal transcriptional activity of S-AR, proposing that SUMOylated AR is resilient to AR antagonists. Additional studies with alternate ARE-reporter constructs were conducted to test for basal activity of S-AR. In absence of exogenous ligand, we observe significant increases in the basal S-AR transactivation of PSA-, GRE- and KLF5-ARE luciferase reporters (Additional file 1: Fig. S4). Consequently, we postulated that S-AR would likely modulate AR-regulated genes including previously identified epithelial-mesenchymal transition genes [30]. In absence of exogenous R1881, S-AR significantly increased several mesenchymal gene transcripts specifically vimentin, fibronectin, transcription factor ZEB1, and MMP2 compared to wild-type AR (Fig. 5d). Activation of ARwt with 10 nM R1881 also enhanced similar mesenchymal gene transcripts. While comparable induction is observed for vimentin and MMP2, S-AR initiates fibronectin and ZEB1 transcription and represses claudin-8 more efficiently than R1881-activated ARwt (Fig. 5d). Collectively, these results highlight that SUMO-PTM supports a ligand-independent hyperactive AR population in TamR-BCa.

### SUMO inhibitor potentiates the anti-metastatic activity of AR’s antagonist Enzalutamide

**Fig. 6:**
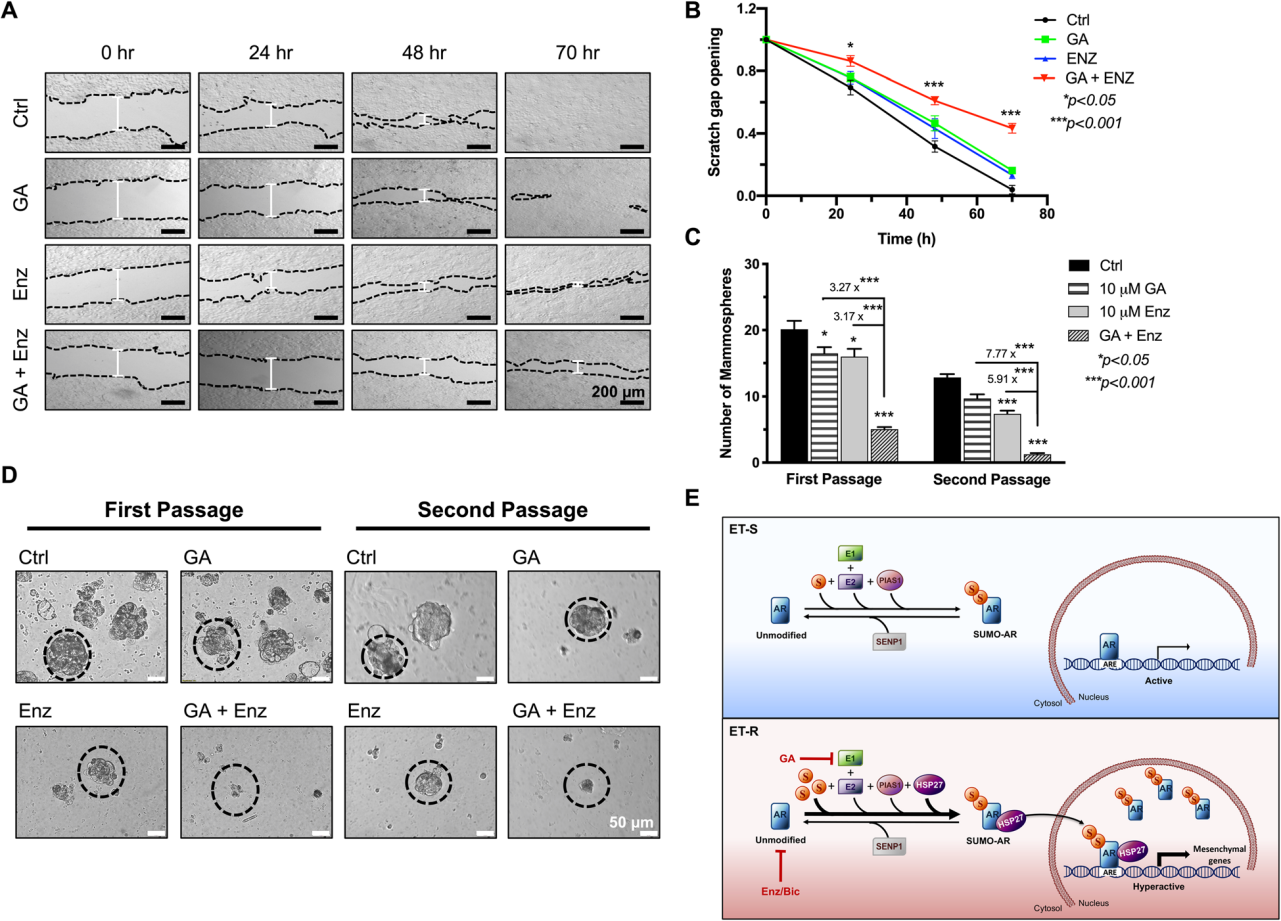
Co-treatments with enzalutamide and SUMO inhibitor decrease the migration and metastatic phenotype of TamR BCa. **a**-**b** Images from scratch wound healing assays indicate that GA + Enz decrease the migration of TamR-7 cells. Black dotted lines define the scratch borders and white lines represent gap width. Remaining scratch gap opening in (**b**) for each treatment at each time point was calculated as a fold relative to the corresponding original gap width at 0 h. Graph is a representation of two independent experiments, each performed in duplicates. **c**-**d** Single-cell suspensions of adherent TamR-7 cells were generated, grown in nonadherent media with indicated treatments and mammosphere images were taken 7 days post treatments. First-generation mammospheres were passaged and dissociated into single cell suspensions and cultured the same way to test for second-generation spheroids. Graph in panel (**c**) represents total mammosphere counts as mean ± SEM from four independent experiments; representative images are shown in (Additional file 1: Fig. S5B). **d** Images illustrate mammosphere size and black-dashed circles highlight size differences between untreated and treated spheroids. Comparisons with control group or between groups, in graphs B and C, were assessed using one-way ANOVA followed by Tukey’s multiple comparison test and significance is illustrated on graphs. **e** Schematic representation of AR regulation by SUMO in ET-S and ET-R BCa. Detailed description is provided in text. Encircled S represents SUMO

We next asked if hyperactive SUMOylated AR supports the migration and metastatic phenotype of TamR-BCa cells. Confluent monolayers of TamR-7 cells were scratched and treated with the SUMO-PTM inhibitor (GA), AR antagonist (Enz), or a combination of GA and Enz (GA + Enz). Concurrent GA + Enz treatment significantly inhibited migration and scratch gap closure of TamR-7 at all time points as compared to control or either drug alone (Fig. 6a-b). We also tested whether suppressed migration of TamR-7 BCa cells by combined GA + Enz therapy is due to reduced proliferation. Percentages of both viable and dead cells of 48-h treated TamR-7 cells were not significantly affected by mono or combined drug treatments when compared to the control group (Additional file 1: Fig. S5A), indicating that GA + Enz inhibits TamR-BCa migration more than growth. Hence, reducing hyperSUMO conditions concurrent with inhibiting AR activation reduces the migration of TamR-7 BCa cells.

We and others observe that TamR-derived MCF-7 BCa cells grow efficiently in non-adherent spheroid cultures than parental MCF-7 cells consistent with their more metastatic phenotype [31, 32]. Hence, we assessed this common anchorage-independent growth and metastatic phenotype of TamR-7 cells. Concurrent GA and Enz or bicalutamide (Bic) treatments reduce the number of mammospheres formed from single-cell suspensions of TamR-7 cells (Fig. 6c and Additional file 1: Fig. S5B, S5D). In addition, the self-renewal capacity of TamR-7 cells is significantly blunted with the GA and Enz treatment as seen with fewer mammospheres forming in second passage studies (Additional file 1: Fig. S5B and 6C). Interestingly, combining GA and Enz produce a synergistic effect on mammosphere counts as compared to control (3.99-fold for 1st generation and 10.47-fold for 2nd generation; Fig. 6c). In addition, treatments with GA + Enz significantly decrease the size of spheroids when compared to control or either drug alone mammospheres (Fig. 6d). Although, 50–100 μm diameter spheroids represent the majority of control and drug alone spheroid population, GA + Enz/Bic treatments produced a population shift with the majority being small-diameter spheroids < 50 μm in first and second passage studies (Additional file 1: Fig. S5C and S5E). Hence, the combination therapy reduces anchorage-independent growth and self-renewal properties of TamR-BCa; both critical for metastasis. Collectively, targeting both unmodified (active) and SUMO-modified (hyperactive) forms of AR could serve as a better approach for treating tamoxifen-resistant breast tumors.

## Discussion

In this study, we report enrichment of SUMO-modified AR with ligand-independent activity in ET-R BCa as supported with the following results. First, while the unmodified AR is present in hormone-sensitive MCF-7 cells, the higher molecular weight SUMO-AR accumulates in cells with acquired (TamR-7) and intrinsic (GI101A and GILM2) resistance to tamoxifen endocrine therapy, and long-term estrogen deprived cells (Fig. 1c). Second, upregulation of SUMO in hormone-sensitive MCF-7 promotes chromatin accumulation of AR analogous to conventional androgen-activated AR (Fig. 4c). Third, also like canonical ligand-activated AR, SUMO-bound AR initiates transcription of ARE-expressing luciferase reporter (Fig. 5b and Fig. S4). Finally, AR antagonist Enz reduces ET-R BCa cell migration and mammosphere formation (Fig. 6) demonstrating that basal AR activity supports aberrant metastatic properties of the drug-resistant cell line.

Clearly tamoxifen therapy impacts how AR directs disease progression; unlike the general HR+ BCa patient population, high AR in patients receiving tamoxifen favors a worse prognosis (Fig. 1a-b). In the tamoxifen-treated patient population, the hazard ratio is further potentiated with concurrent induction of the SUMO isoforms (Fig. 2c). Consistently the global SUMOylome is also upregulated in the TamR cell lines as compared to non-cancerous or hormone-sensitive BCa cells (Fig. 2d-e). A previous study shows that Myc-dependent BCa require specifically the E1-SAE2 dimer component for tumor growth [33]. While we did not observe an SAE2 elevation in ET-R HR+ BCa cells, clearly a need to potentiate the SUMOylome is a shared event for cancer progression. Consistently, others propose inhibiting global SUMO-PTM with GA or less selective Anacardic acid as promising therapy for the another BCa subtype TNBC [34, 35]. In our studies, HR+ BCa cells resistant to ET are responsive to the inhibitory actions of GA (Fig. 6). Like basal ER negative, ET-R ERα + BCa cells are more mesenchymal than stagnant MCF7, suggesting that accumulation of SUMOylated proteins could drive the metastatic phenotype in multiple BCa subtypes. In the present study, we demonstrate that SUMO-AR supports the transcriptome profile required to initiate this phenotype.

For AR, induction of SUMO is not sufficient for AR SUMOylation. In our cell-free system, we demonstrate that the SUMO-E3 ligase Hsp27 is required for SUMO-PTM of AR (Fig. 3d). In vivo, high SUMO levels, analogous to endogenous levels in TamR-BCa, support greater binding between AR and Hsp27 than another established AR SUMO E3 ligase PIAS1 [25, 28] (Fig. 3a, b, and S2A). Hsp27 exhibits SUMO-E3 ligase enzymatic activity for limited protein substrates specifically HSF1 [36] and CFTR mutant F508del [37]. Consistent with SUMO E3 ligase activity for these other substrates, Hsp27 concurrently binds SUMO, SUMO-E2 Ubc9, and substrate-AR (Fig. 3c) to drive AR SUMO-conjugation kinetics (Fig. 3d). Unlike PIAS1 [25, 28], Hsp27 supports AR SUMOylation in the absence AR-ligand (Fig. 3). These findings present an additional regulatory control mechanism based on PTM to the canonical substrate-chaperone relationship between AR and Hsp27 established previously [27, 38].

AR agonist DHT stimulates transcription of mesenchymal genes and metastasis of MCF7 cells in xenograft studies [30]. In MCF7 cells, SUMO-bound AR functions analogous to ligand-activated AR. Specifically, SUMO-AR initiates transcription of multiple mesenchymal-driving genes equivalent to or greater than R1881-activated AR (Fig. 5d). Yet surprisingly, transcriptional activity of canonical ligand-activated but not SUMO-bound AR responded to Enz treatments. Present results show that lowering global SUMOylation potentiates the effectiveness of AR antagonists and may have greater impact in inhibiting TamR-BCa migration more than growth (Fig. 6b; Additional file 1: Fig. S5A). Mesenchymal TamR BCa enriched in nonadherent spheroid conditions are more responsive to Enz/GA combinatorial therapy (Fig. 6c). While raising evidence advocates the use of AR antagonists clinically for advanced HR + -BCa [39, 40], Enz in combination with exemestane failed to improve the PFS of HR+ postmenopausal women with prior endocrine therapy but was successful for those who did not receive it [9]. It is intriguing to speculate that these clinical results are due to differences in a mixed AR population (unmodified and SUMOylated AR). If the majority of AR is being highly modified, then Enz will antagonize the ligand activated unmodified AR but not the hyperactive modified AR which functionally does not depend entirely on the ligand.

## Conclusion

Collectively and as illustrated in Fig. 6e, SUMOylation of AR in ET-S cells is dynamic and reversible. However, a sustained hyperSUMO environment, observed in ET-R but not in ET-S cells, favors AR-SUMOylation and enhances its interaction with Hsp27. Concomitant rise in the levels of nuclear SUMOylated AR available for chromatin binding results in AR-mediated transcriptional activation and induction of genes promoting metastasis. Hence, a better approach in targeting various AR populations in ET-R BCa, as the unmodified (active) and SUMO-modified (hyperactive) forms of AR, could be achieved by combining AR-targeted therapies (as Enz or Bic) with SUMOylation inhibitors (as GA).

Our data suggest that dual targeting of AR and SUMO might have clinical and therapeutic relevance in the ET-R HR+ BCa subset of breast cancer. Therefore, increased global SUMOylation and in particular SUMO-modified AR may be a potential predictive biomarker useful to stratify HR+ BCa patients that are most likely to benefit from combining AR antagonists with SUMO inhibitors.

## Supplementary information

**Additional file 1. **Supplemental materials and methods: in vitro SUMOylation; RT-PCR; Luciferase reporter assays; PLA, mammosphere studies; scratch assays; transcriptomic data analysis. **Table S1.** List of primer sequences used for the detection of transcripts. Supplemental figures and figure legends: **Figure S1.** Elevated levels of SUMO isoforms and HSP27 correlate with high probability of metastasis in ET-treated HR+ BCa patients. **Figure S2.** HyperSUMO conditions promotes AR SUMOylation and enhances its interaction with Hsp27. **Figure S3.** SUMO stabilizes AR and reduces its proteasomal degradation. **Figure S4.** SUMO stimulates basal AR transcriptional activity regardless of the AR-luciferase reporter construct. **Figure S5.** Concurrent targeting of SUMO-modified and unmodified AR decreases TamR-7 BCa growth in 3D cultures.

## Data Availability

Not applicable.

## Abbreviations

AI: aromatase inhibitor
AR: androgen receptor
ARE: androgen response elements
BCa: breast cancer
DMFS: distant-metastasis free survival
Enz: enzalutamide
HR+: hormone receptor-positive
GA: ginkgolic acid
Hsp27: heat shock protein 27
HR+: hormone receptor positive
ET-R: endocrine therapy-resistant
ET-S: endocrine therapy-sensitive
PFS: progression-free survival
PIAS1: Protein inhibitor of activated STAT
PTM: posttranslational modifications
PLA: Proximity Ligation Assay
SENP1: SUMO specific peptidase 1
SUMO: Small Ubiquitin Like Modifier
S-AR: SUMO-fused-AR
TamR: tamoxifen resistant
TamS: tamoxifen sensitive
ARwt: wild-type AR
