# Supplementary material for: Constitutively active androgen receptor supports the metastatic phenotype of endocrine-resistant hormone receptor-positive breast cancer
Source: Cell Commun Signal. 2020 Sep 18;18:154. doi: 10.1186/s12964-020-00649-z (PMC7501670; doi:10.1186/s12964-020-00649-z)
Supplement: Supplementary file 2 — Additional file 1. Supplemental materials and methods: in vitro SUMOylation; RT-PCR; Luciferase reporter assays; PLA, mammosphere studies; scratch assays; transcriptomic data analysis. Table S1. List of primer sequences used for the detection of transcripts. Supplemental figures and figure legends: Figure S1. Elevated levels of SUMO isoforms and HSP27 correlate with high probability of metastasis in ET-treated HR+ BCa patients. Figure S2. HyperSUMO conditions promotes AR SUMOylation and enhances its interaction with Hsp27. Figure S3. SUMO stabilizes AR and reduces its proteasomal degradation. Figure S4. SUMO stimulates basal AR transcriptional activity regardless of the AR-luciferase reporter construct. Figure S5. Concurrent targeting of SUMO-modified and unmodified AR decreases TamR-7 BCa growth in 3D cultures. [file 12964_2020_649_MOESM2_ESM.zip › 2_Final Additional file.docx]

**Constitutively Active Androgen Receptor Supports**

**the Metastatic Phenotype of Endocrine-Resistant Hormone Receptor-Positive Breast Cancer**

Shaymaa Bahnassy^1^, Hariprasad Thangavel^2^, Maram Quttina^1^, Ashfia Khan^1^, Dhanya Dhanyalayam^1^, Joan Ritho^3^, Samaneh Karami^1^, Jing Ren^4^, and Tasneem Bawa-Khalfe^1*^

^1^Center for Nuclear Receptors & Cell Signaling, Department of Biology & Biochemistry, University of Houston, Houston, Texas 77204, USA.

^2^Department of Pharmacy Practice and Translational Research, College of Pharmacy, University of Houston, Houston, Texas 77204, USA.

^3^Department of Biology, Stanford University, Stanford, California 94305, USA.

^4^Center for Precision Medicine, Department of Medicine, University of Missouri, Columbia, MO 65212, USA.

Authors’ e-mail addresses (in order): sabahnas@central.uh.edu; hariprasadslm@gmail.com; mquttina@central.uh.edu; akhan70@central.uh.edu; dhanyalayamd@gmail.com; jritho@stanford.edu; skarami@cougarnet.uh.edu; renjing5706@yahoo.com; tbawa-khalfe@uh.edu

*Corresponding Author: Tasneem Bawa-Khalfe; Center for Nuclear Receptor & Cell Signaling, Department of Biology & Biochemistry, University of Houston; 3517 Cullen Blvd, SERC Bldg, Rm 3010, Houston, TX 77204-5056; Phone: 713-743-4288; Email: tbawa-khalfe@uh.edu

**SUPPLEMENTAL MATERIALS and METHODS**

*In vitro* SUMOylation Assays

Recombinant SAE1/SAE2 (SUMOLink, Active Motif), purified GST-tagged proteins (GST-SUMO-2 and GST-Ubc9), and recombinant Hsp27 (Abcam) were all used in the *in vitro* SUMOylation assays. Standard GST protein purification was performed with Glutathione Spin Columns containing GST-Bind resin (ThermoFisher Scientific). *In vitro* SUMOylation assay was performed as described previously ([1](#_ENREF_1)). Briefly, AR protein isolated from MCF-7 cells was incubated with SAE1/SAE2, Ubc9, and SUMO2 in presence or absence of Hsp27 in a SUMOylation buffer (50 mM Tris, 10 mM MgCl_2_, 1mM DTT, 5 mM ATP) for 3 hrs at 30 ^o^C. SDS loading buffer was added to terminate the SUMOylation reaction and samples were resolved by SDS/PAGE and immunoblotted with specific antibodies.

Proximity Ligation Assay (PLA)

The DuoLink assay kit (Sigma-Aldrich) was used according to manufacturer’s instructions. Briefly, MCF-7 cells were grown on coverslips in 6-well plates then transfected. After 48 hrs from transfection, cells were fixed, permeabilized then blocked. Samples were incubated with primary antibodies AR (sc-816, Santa Cruz Biotechnology) and SUMO2/3 (8A2, Kerafast) and subsequently incubated with secondary antibodies linked to PLA probes. Ligation and amplification were followed by DAPI mounting to visualize the nuclei and images were captured by Nikon Eclipse Ti2. The total number of puncta, representing AR/SUMO3 complexes, per nuclei were quantified using Image J (NIH).

Luciferase Reporter Assays

To generate the SUMOylated AR mimetic, AR insert was amplified with the following forward (5’-ATGGAAGTGCAGTTAGGGCTG-3’ and 5’-TTTCCCCGGCTTAAGCAGCT-3’) and reverse (5’-TCACTGGGTGTGGAAATAGATGGG- 3’) primers. Next, the pcDNA3/HA-SUMO3 plasmid, described in ([2](#_ENREF_2)), was linearized and AR was subsequently cloned into the vector using the In-Fusion HD Cloning Kit (Clontech). To confirm that the AR gene has been inserted correctly, sequencing and restriction digests, with BamHI and XbaI, were conducted. Four different AR-regulated luciferase reporter constructs were used. These include our ARE_3_-probasin-, GRE_2_E1b-, PSA- and pGL4.26-KLF5-enhancer-luciferase plasmids. Details of these luciferase plasmids have been described in previous publications ([3-6](#_ENREF_3)). MCF-7 cells were seeded into 24-well plates using phenol red-free media supplemented with 2% charcoal-stripped fetal bovine serum (CSS). After 48 hrs, cells were co-transfected with firefly and renilla luciferase along with ARwt or SUMO3-fused-AR. Equal amounts of transfected plasmids were ensured by the addition of empty vector (EV). On the next day, cells were either unstimulated or treated with 10 nM R1881 for 24 h in the same media described before. Lysates were generated, the dual-luciferase reporter assay system (Promega) was used to measure firefly and renilla activities according to manufacturer’s instructions and luminescence levels were read on a microplate reader (VICTOR X4, PerkinElmer). Three independent experiments were conducted, and each experiment was performed in triplicates.

Real-time PCR

Total RNA of biological triplicates was extracted from cells with PureLink RNA Mini Kit (ThermoFisher Scientific) and converted to cDNA with iScript cDNA Synthesis Kit (BioRad) according to manufacturer’s instructions. Expression of target genes with specific primers (Additional file 1: Table S1; IDT) was measured by RT-qPCR using the iTaq Universal SYBR Green Supermix (BioRad) and 7500 Fast Real-Time PCR System (Applied Biosystems). Data was normalized to the reference gene and analyzed by the ΔΔCT method.

Trypan Blue Exclusion Assay

TamR-7 cells were seeded at a density of 2*10^4^ cells/well in 24-well plates. After cell attachment, cells were either treated with enzalutamide (MedChem Express), ginkgolic acid (C15:1, Sigma-Aldrich) or a combination of both. After 48 hours of treatment, cells were trypsinized and counted by the trypan blue staining method using Cellometer Vision CBA (Nexcelom). Cell viability and death percentages were then calculated and compared between groups.

Scratch wound healing assays

TamR-7 cells were seeded into 12-well plates at a seeding density of 70,000 cells/well. On the next day, cells became confluent and a scratch was made using a sterile 200 µl pipette tip. After washing with PBS, cells were further cultured in media containing vehicle, 10 µM Enz, 10 µM GA or both. Images were acquired and the gap width of scratch was measured at 0, 24, 48 and 70 hrs, and compared with the initial gap size at 0 hr. The % scratch gap closure was calculated and graphed.

Mammosphere Studies

Single cell suspensions of TamR cells were generated and seeded into ultra-low attachment plates (Corning) at a density of 30,000 cells/ml. Cells were grown in non-adherent conditions for seven days using MammoCult media (Stemcell) supplemented with Heparin (Sigma-Aldrich), hydrocortisone (Sigma-Aldrich), mammoCult proliferation supplement (Stemcell), methylcellulose (Sigma-Aldrich) and penicillin/streptomycin (ThermoFisher Scientific). The media contained treatments of AR antagonists, ginkgolic acid or a combination of both. One milliliter of fresh media was added to each well every two days without removing the old media. To test for self-renewal properties, first generation mammospheres were passaged and dissociated into single cells then 30,000 cells/ml were reseeded. Images were captured, mammsopheres were counted and their diameters were measured using Image J (NIH).

**Table S1:** List of primer sequences used for the detection of transcripts.

| Gene | Primer Sequence | Reference |
| --- | --- | --- |
| SUMO-1 | **Forward:** 5’-AGCAGTGAGATTCACTTCAAAGTG-3’  **Reverse:** 5’-TCTGACCCTCAAAGAGAAACC-3’ |  |
| SUMO-2 | **Forward:** 5’-GGATTGTCAATGAGGCAGATCAG-3’  **Reverse:** 5’-CCGTCTGCTGTTGGAACACATC-3’ |  |
| SUMO-3 | **Forward**: 5’-GGCTTGTCAATGAGGCAGATCAG-3’  **Reverse:** 5’-CTGCTGGAACACGTCGATGGTG-3’ |  |
| SAE1 | **Forward:** 5’-AGGACTGACCATGCTGGATCAC-3’  **Reverse:** 5’-CTCAGTGTCCACCTTCACATCC-3’ |  |
| UBA2 (SAE2) | **Forward:** 5’-AGAGGTGACTGTGCGGCTGAAT-3’  **Reverse:** 5’-GGACATCTGGTGCTACCATAGC-3’ |  |
| UBE2I  (UBC9) | **Forward:** 5’-ATCCAAGACCCAGCTCAAGCAG-3’  **Reverse:** 5’-TTGACGATGCCACAAGGTCGCT-3’ |  |
| PIAS1 | **Forward:** 5’-TAAGGAGGATGGCACTTGGGCA-3’  **Reverse:** 5’-TGAGACGCTACCTGATGCTCCA-3’ |  |
| HSPB1  (HSP27) | **Forward:** 5’-TCCCTGGATGTCAACAACTTC-3’  **Reverse:** 5’- TCTCCACCACGCCATCCT-3’ | ([7](#_ENREF_7)) |
| SENP1 | **Forward:** 5’-ATCAGGCAGTGAAACGTTGGAC-3’  **Reverse:** 5’-GCAGGCTTCATTGTTTATCCCA-3’ | ([8](#_ENREF_8)) |
| β-actin | **Forward:** 5’-TGTACGCCAACACAGTGCTG-3’  **Reverse:** 5’-GCTGGAAGGTGGACAGCGA-3’ |  |
| Vimentin (VIM) | **Forward:** 5’-TACAGGAAGCTGCTGGAAGGCG-3’  **Reverse:** 5’- TGGCAGAGGCAGAGAAATCCTGC-3’ | ([9](#_ENREF_9)) |
| Fibronectin  (FN1) | **Forward:** 5’-CCGCCGAATGTAGGACAAGA-3’  **Reverse:** 5’-TGCCAACAGGATGACATGAAA-3’ | ([9](#_ENREF_9)) |
| ZEB1 | **Forward:** 5’-GCACCTGAAGAGGACCAGAG-3’  **Reverse:** 5’-TGCATCTGGTGTTCCATTTT-3’ | ([10](#_ENREF_10)) |
| ZEB2 | **Forward:** 5’-TTTCAGGGAGAATTGCTTGA-3’  **Reverse:** 5’-CACATGCATACATGCCACTC-3’ | ([11](#_ENREF_11)) |
| MMP9 | **Forward:** 5’-CCGAGCTGACTCGACGGTGATGG-3’  **Reverse:** 5’-GAGGTGCCGGATGCCATTCACGTC-3’ | ([12](#_ENREF_12)) |
| MMP2 | **Forward:** 5’-CCCCAAAACGGACAAAGAG-3’  **Reverse:** 5’-CACGAGCAAAGGCATCATCC-3’ | ([13](#_ENREF_13)) |
| E-cadherin (CDH1) | **Forward:** 5’- GAGAGGAATCCAAAGCCTCAGGT-3’  **Reverse:** 5’- CTGGTTATCCATGAGCTTGAGAT-3’ | ([9](#_ENREF_9)) |
| Claudin 8 (CLD8) | **Forward:** 5’-CGTGAGGCAGGCTAACATCA-3’  **Reverse:** 5’-AGCAGCACACATCAGTCCTC-3’ | ([14](#_ENREF_14)) |
| 18 S | **Forward:** 5’-AGAAACGGCTACCACATCCA-3’  **Reverse:** 5’-CACCAGACTTGCCCTCCA-3’ |  |

**SUPPLEMENTAL FIGURES and FIGURE LEGENDS

**

**Figure S1. Elevated levels of SUMO isoforms and HSP27 correlate with high probability of metastasis in ET-treated HR+ BCa patients. (A)** Transcript levels of Hsp27/HSPB1 are significantly higher in TamR-7 BCa cells. The graph represents fold-change in 2^ΔΔCt^ values of 3 independent experiments and statistical significance in raw ΔCt values using Student’s t-test. **(B-D)** Kaplan–Meier distant metastasis free survival curves were generated from the KM Plotter database for Tam-treated HR+-BCa patients and separate high and low gene expressions for: **(B)**all SUMO isoforms;**(C-D)** Hsp27/HSPB1 and PIAS1 (E3 ligases). The hazard ratio at 95% confidence and the logrank P values are enlarged within the plots.

**
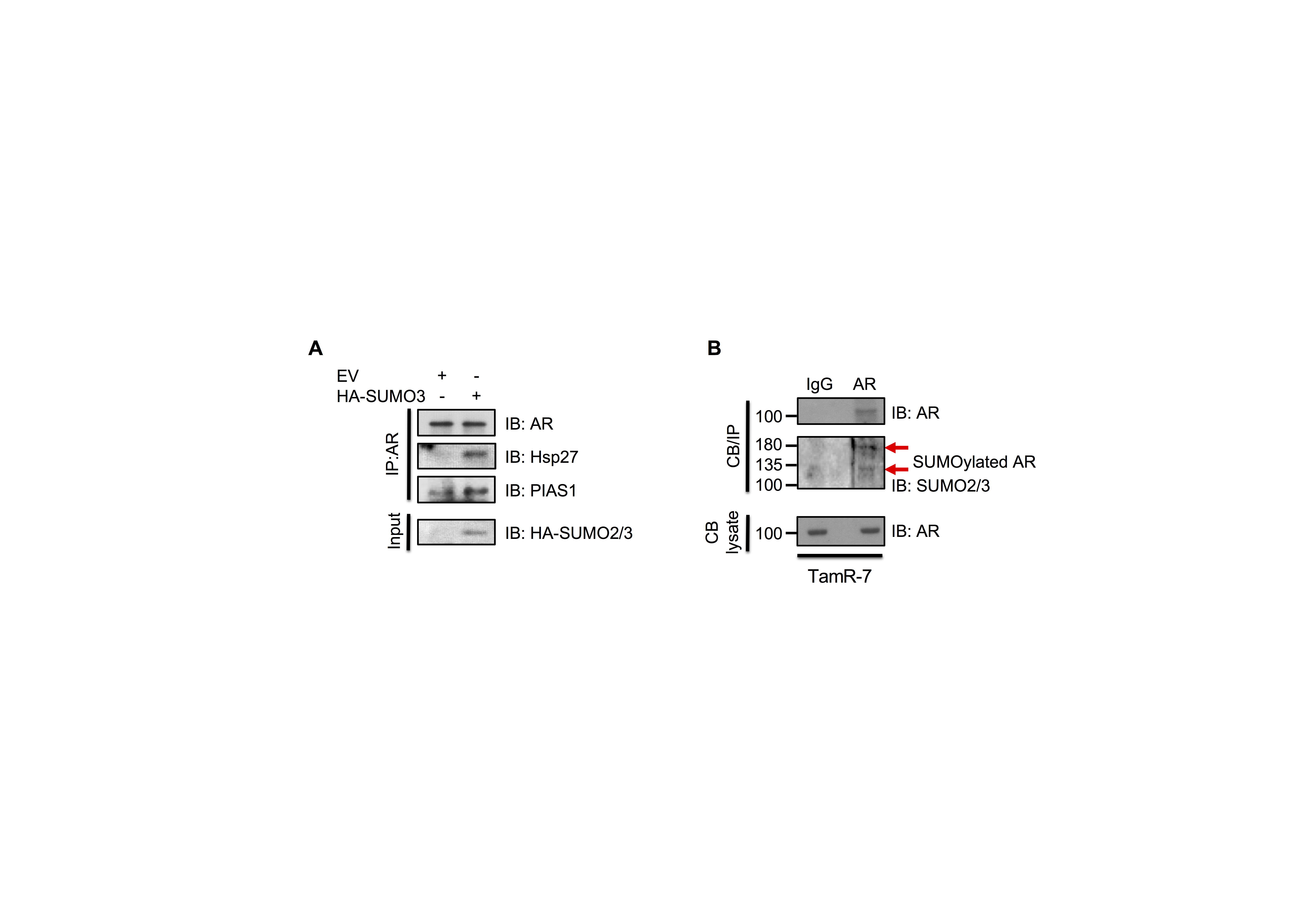
**

**Figure S2. HyperSUMO conditions promotes AR SUMOylation and enhances its interaction with Hsp27.** Endogenous AR was immunoprecipitated from: **(A)** MCF-7 cells that were transiently transfected with EV or HA-SUMO3 and **(B)** from chromatin-bound (CB) fractions of TamR-7 cells. Proteins were subsequently resolved by SDS-PAGE and immunoblotted with specific antibodies. Arrows in (B) indicate SUMO-2/3 conjugates of AR.



**Figure S3. SUMO stabilizes AR and reduces its proteasomal degradation.** Cycloheximide (CHX) experiments show that AR’s rate of degradation is slower within the hyperSUMO environment of TamR-7 versus parental MCF-7 cells **(A)** and that endogenous AR is more stable in MCF-7 cells that overexpress SUMO-3 **(B)**. Cells were untransfected in (A) or transfected with either empty vector (EV) or HA-SUMO3 (B). On the next day, cells were treated with 10 µM CHX for the indicated time periods and proteins were detected by immunoblotting using anti-AR, anti-HA and anti-GAPDH antibodies. Band intensities were quantified by densitometry using ImageJ. Graphs in panels (A) and (B) illustrate the remaining amounts of AR normalized to the loading control from at least three independent experiments. Statistical significance of *p<0.05 and **p<0.01 were obtained by Student’s t-test. **(C)** Mimicking the TamR-7 conditions, by overexpressing AR and SUMO-3 reduces total ubiquitylation of AR in MCF-7 cells. AR pull-down was analyzed by western blot analysis using specific antibodies; the bracket indicates ubiquitylated AR.

**
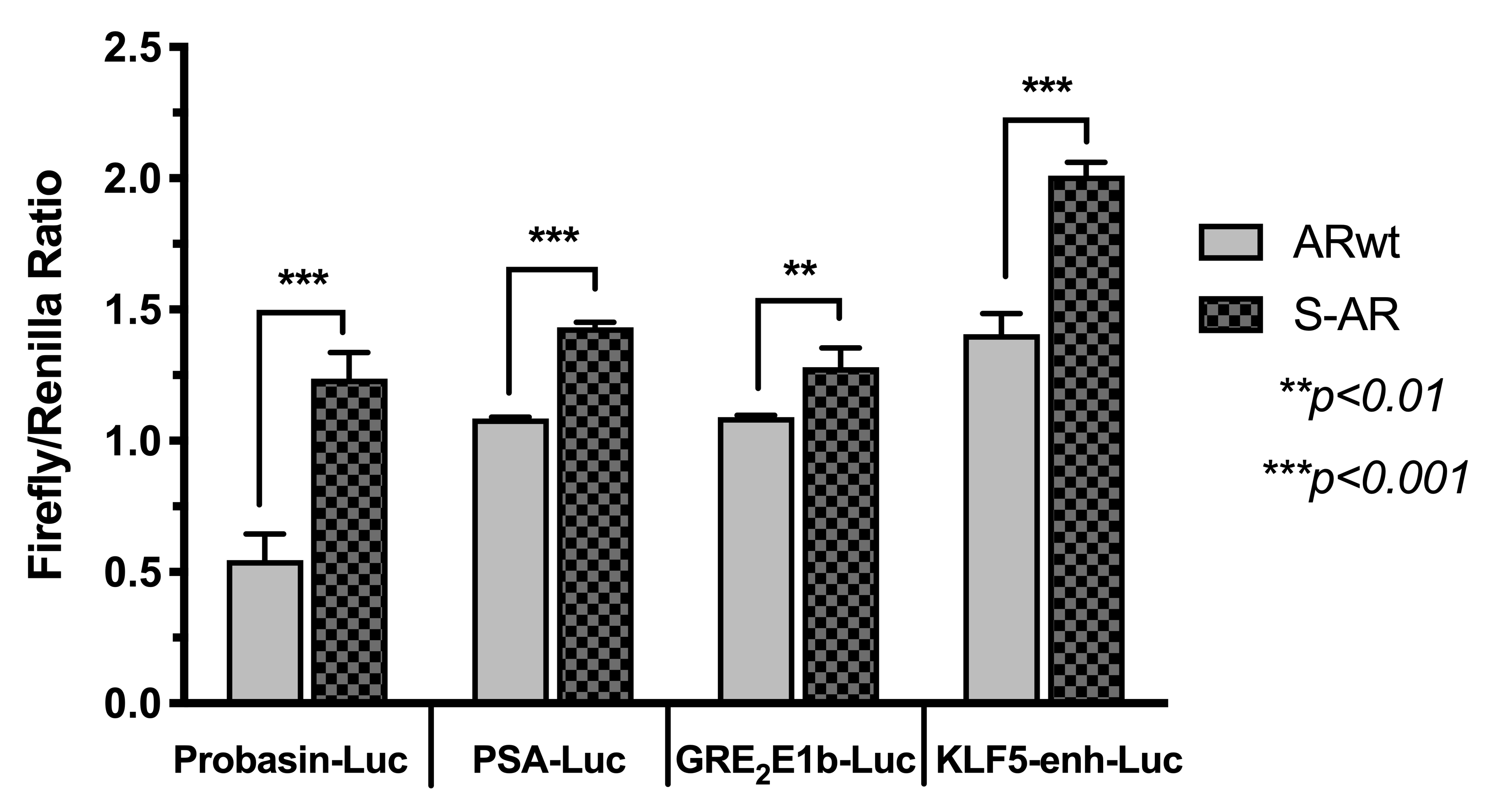
**

**Figure S4. SUMO stimulates basal AR transcriptional activity regardless of the AR-luciferase reporter construct.** MCF-7 cells were grown in phenol-red free DMEM media with 2% CSS for 48 hours. Various luciferase reporter constructs (probasin, PSA- or KLF5-enh-Luc) were co-transfected with Renilla luciferase together with either ARwt or SUMO-fused AR (S-AR). After 24 hrs, cells were harvested and assayed for luciferase activity. Data represent fold-change in firefly/renilla ratios ± SEM of three independent experiments, each performed in triplicates. **p<0.01 and ***p<0.001 indicate significant changes from the corresponding ARwt group using Student’s *t*-test.


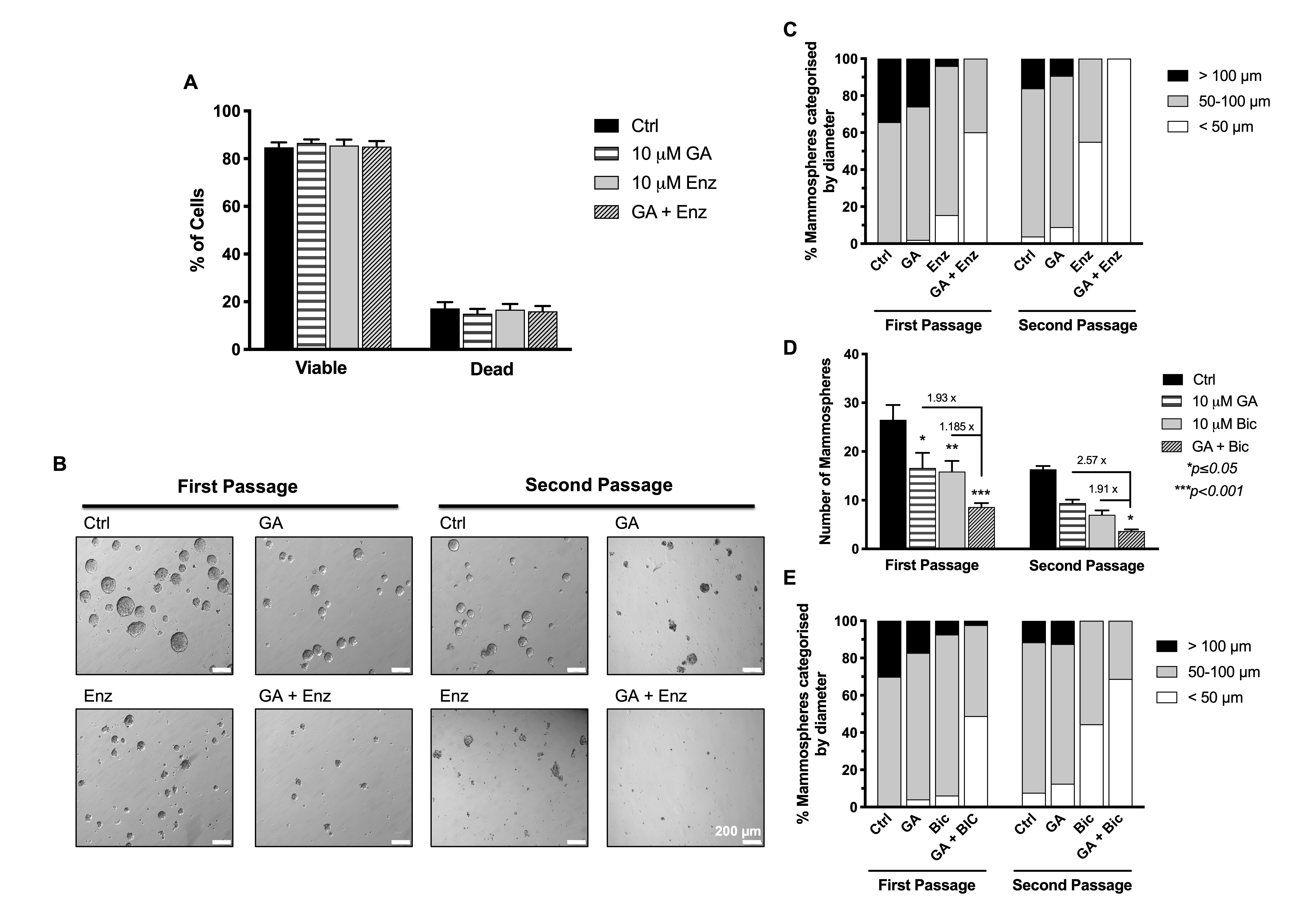


**Figure S5. Concurrent targeting of SUMO-modified and unmodified AR decreases TamR BCa growth in 3D cultures. (A)** Cell counts of TamR-7 were measured by Cellometer using the Trypan blue exclusion assay after 48 hours of the indicated treatments. Percentages of live and dead cells were quantified and graphed as mean ± SEM from three independent experiments, each performed in quadruplicates. **(B-E)** First- and second-generation spheroids of TamR-7 were treated with 10 µM of the indicated treatments. **(B)** Representative images of mammosphere counts quantified and graphed in Fig. 6A. Graphs in panels **(C)** and **(E)** represent percentage of mammospheres based on their diameters while **(D)** represents mammosphere counts from two independent experiments. Comparisons with the control group were assessed using one-way ANOVA followed by Tukey’s multiple comparison test and statistical significance is shown on graphs.

**REFERENCES**

1. Lin FM, Kumar S, Ren J, Karami S, Bahnassy S, Li Y, et al. SUMOylation of HP1alpha supports association with ncRNA to define responsiveness of breast cancer cells to chemotherapy. Oncotarget. 2016;7(21):30336-49.

2. Kamitani T, Nguyen HP, Kito K, Fukuda-Kamitani T, Yeh ETH. Covalent Modification of PML by the Sentrin Family of Ubiquitin-like Proteins. Journal of Biological Chemistry. 1998;273(6):3117-20.

3. Frigo DE, Sherk AB, Wittmann BM, Norris JD, Wang Q, Joseph JD, et al. Induction of Krüppel-Like Factor 5 Expression by Androgens Results in Increased CXCR4-Dependent Migration of Prostate Cancer Cells in Vitro. Molecular Endocrinology. 2009;23(9):1385-96.

4. Agoulnik IU, Krause WC, Bingman WE, 3rd, Rahman HT, Amrikachi M, Ayala GE, et al. Repressors of androgen and progesterone receptor action. J Biol Chem. 2003;278(33):31136-48.

5. Cheng J, Wang D, Wang Z, Yeh ET. SENP1 enhances androgen receptor-dependent transcription through desumoylation of histone deacetylase 1. Mol Cell Biol. 2004;24(13):6021-8.

6. Blessing AM, Rajapakshe K, Reddy Bollu L, Shi Y, White MA, Pham AH, et al. Transcriptional regulation of core autophagy and lysosomal genes by the androgen receptor promotes prostate cancer progression. Autophagy. 2017;13(3):506-21.

7. Vahid S, Thaper D, Gibson KF, Bishop JL, Zoubeidi A. Molecular chaperone Hsp27 regulates the Hippo tumor suppressor pathway in cancer. Sci Rep. 2016;6:31842.

8. Xu Y, Zuo Y, Zhang H, Kang X, Yue F, Yi Z, et al. Induction of SENP1 in endothelial cells contributes to hypoxia-driven VEGF expression and angiogenesis. J Biol Chem. 2010;285(47):36682-8.

9. Karami S, Lin FM, Kumar S, Bahnassy S, Thangavel H, Quttina M, et al. Novel SUMO-Protease SENP7S Regulates beta-catenin Signaling and Mammary Epithelial Cell Transformation. Sci Rep. 2017;7:46477.

10. Drake JM, Strohbehn G, Bair TB, Moreland JG, Henry MD. ZEB1 enhances transendothelial migration and represses the epithelial phenotype of prostate cancer cells. Mol Biol Cell. 2009;20(8):2207-17.

11. Nam E-H, Lee Y, Park Y-K, Lee JW, Kim S. ZEB2 upregulates integrin α5 expression through cooperation with Sp1 to induce invasion during epithelial–mesenchymal transition of human cancer cells. Carcinogenesis. 2012;33(3):563-71.

12. Struckmann K, Mertz K, Steu S, Storz M, Staller P, Krek W, et al. pVHL co-ordinately regulates CXCR4/CXCL12 and MMP2/MMP9 expression in human clear-cell renal cell carcinoma. The Journal of Pathology. 2008;214(4):464-71.

13. Partridge JJ, Madsen MA, Ardi VC, Papagiannakopoulos T, Kupriyanova TA, Quigley JP, et al. Functional analysis of matrix metalloproteinases and tissue inhibitors of metalloproteinases differentially expressed by variants of human HT-1080 fibrosarcoma exhibiting high and low levels of intravasation and metastasis. J Biol Chem. 2007;282(49):35964-77.

14. Sutinen P, Malinen M, Heikkinen S, Palvimo JJ. SUMOylation modulates the transcriptional activity of androgen receptor in a target gene and pathway selective manner. Nucleic Acids Research. 2014;42(13):8310-9.
