# Supplementary figures and images for: Constitutively active androgen receptor supports the metastatic phenotype of endocrine-resistant hormone receptor-positive breast cancer
Source: Cell Commun Signal. 2020 Sep 18;18:154. doi: 10.1186/s12964-020-00649-z (PMC7501670; doi:10.1186/s12964-020-00649-z)

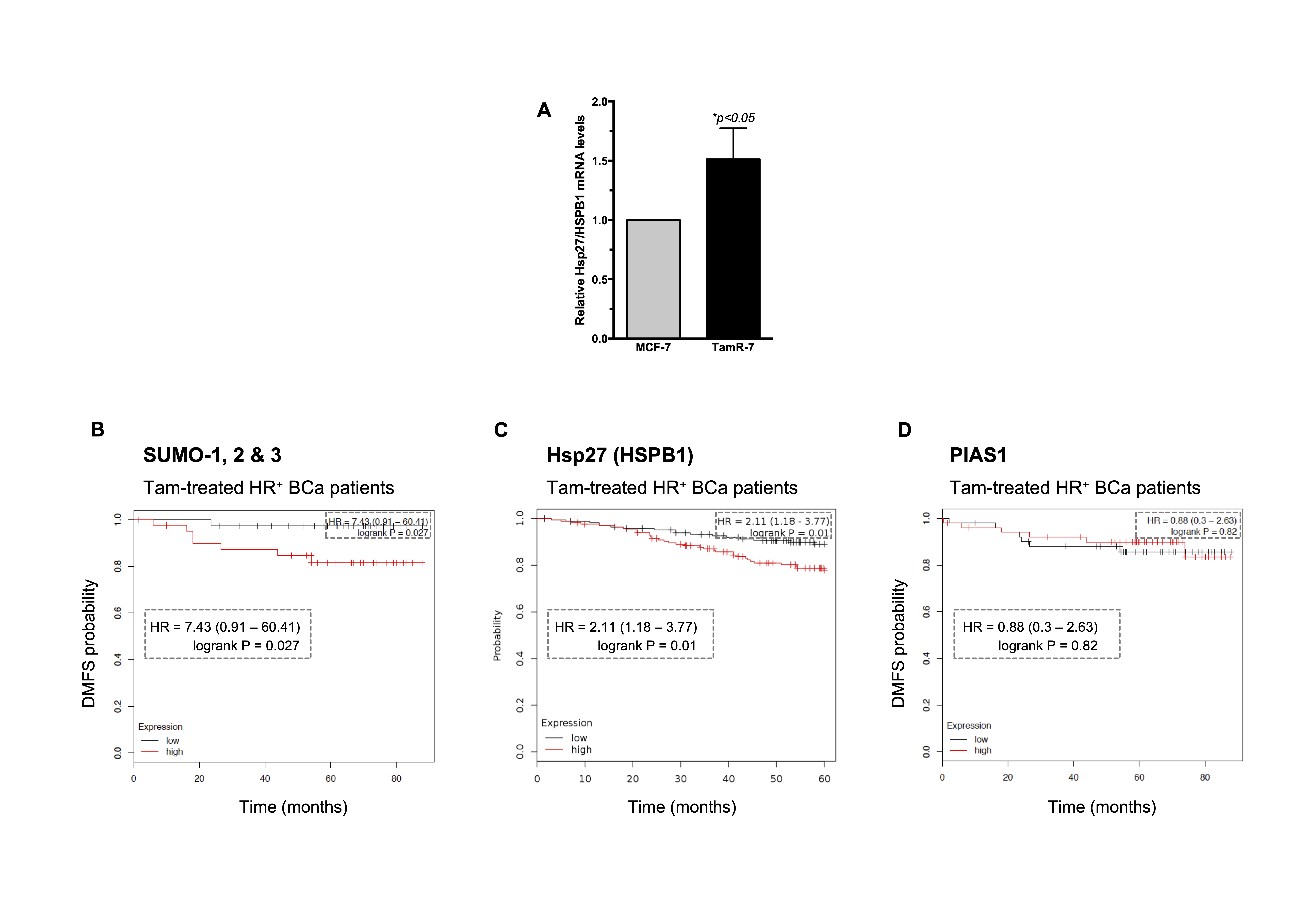

Supplement: Supplementary file 2 — Additional file 1. Supplemental materials and methods: in vitro SUMOylation; RT-PCR; Luciferase reporter assays; PLA, mammosphere studies; scratch assays; transcriptomic data analysis. Table S1. List of primer sequences used for the detection of transcripts. Supplemental figures and figure legends: Figure S1. Elevated levels of SUMO isoforms and HSP27 correlate with high probability of metastasis in ET-treated HR+ BCa patients. Figure S2. HyperSUMO conditions promotes AR SUMOylation and enhances its interaction with Hsp27. Figure S3. SUMO stabilizes AR and reduces its proteasomal degradation. Figure S4. SUMO stimulates basal AR transcriptional activity regardless of the AR-luciferase reporter construct. Figure S5. Concurrent targeting of SUMO-modified and unmodified AR decreases TamR-7 BCa growth in 3D cultures. [file 12964_2020_649_MOESM2_ESM.zip › Fig S1.tiff]

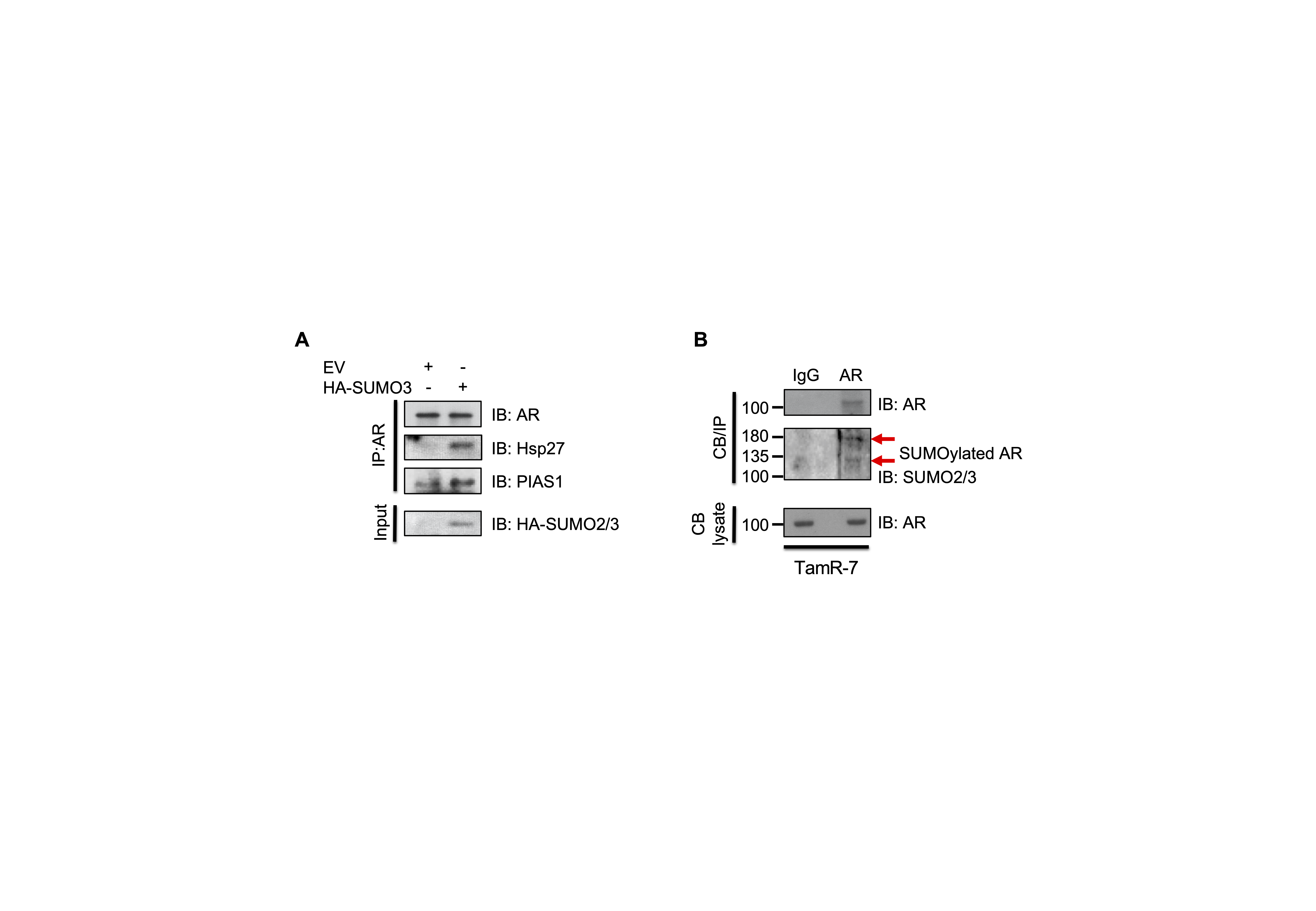

Supplement: Supplementary file 2 — Additional file 1. Supplemental materials and methods: in vitro SUMOylation; RT-PCR; Luciferase reporter assays; PLA, mammosphere studies; scratch assays; transcriptomic data analysis. Table S1. List of primer sequences used for the detection of transcripts. Supplemental figures and figure legends: Figure S1. Elevated levels of SUMO isoforms and HSP27 correlate with high probability of metastasis in ET-treated HR+ BCa patients. Figure S2. HyperSUMO conditions promotes AR SUMOylation and enhances its interaction with Hsp27. Figure S3. SUMO stabilizes AR and reduces its proteasomal degradation. Figure S4. SUMO stimulates basal AR transcriptional activity regardless of the AR-luciferase reporter construct. Figure S5. Concurrent targeting of SUMO-modified and unmodified AR decreases TamR-7 BCa growth in 3D cultures. [file 12964_2020_649_MOESM2_ESM.zip › Fig S2.tiff]

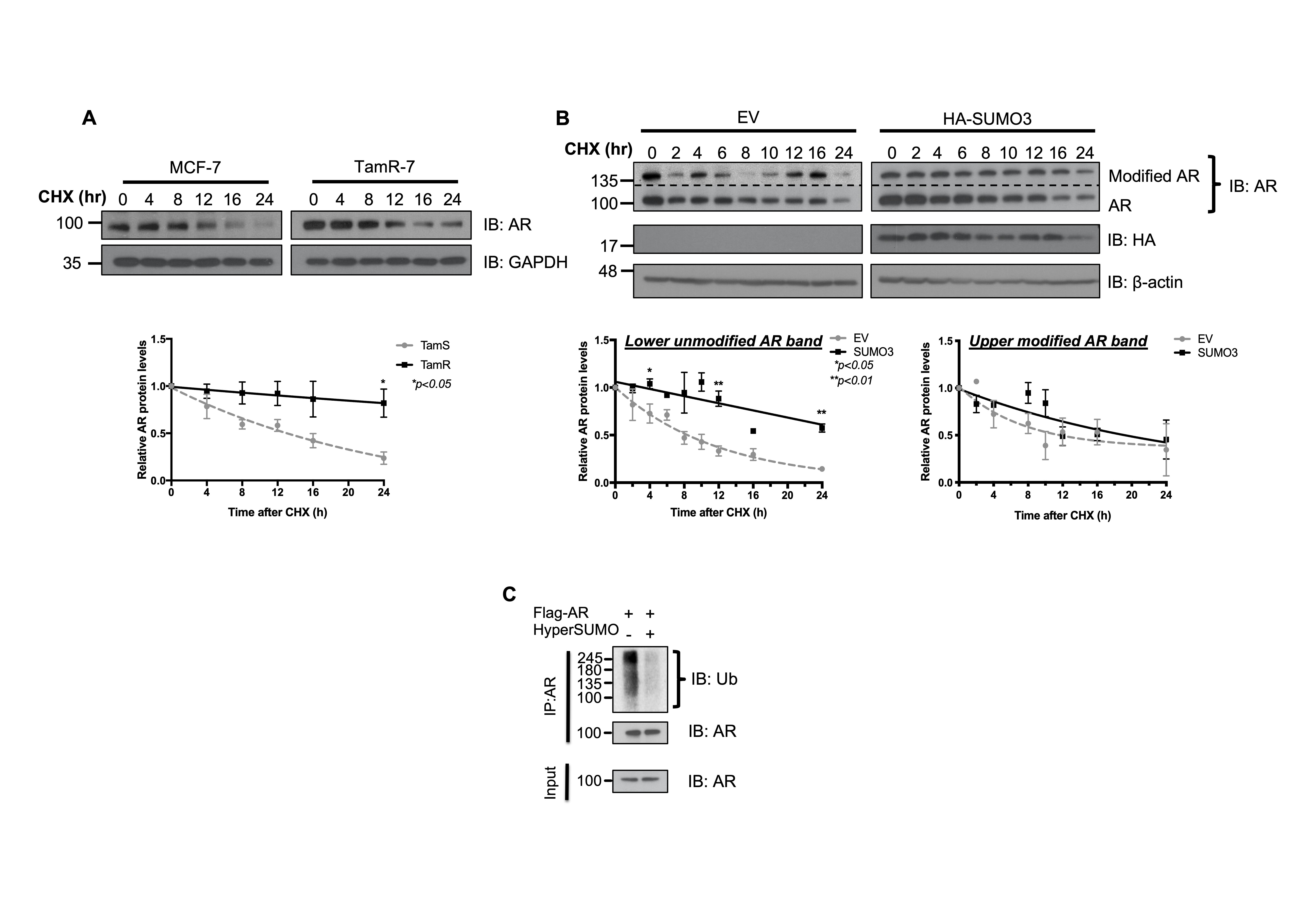

Supplement: Supplementary file 2 — Additional file 1. Supplemental materials and methods: in vitro SUMOylation; RT-PCR; Luciferase reporter assays; PLA, mammosphere studies; scratch assays; transcriptomic data analysis. Table S1. List of primer sequences used for the detection of transcripts. Supplemental figures and figure legends: Figure S1. Elevated levels of SUMO isoforms and HSP27 correlate with high probability of metastasis in ET-treated HR+ BCa patients. Figure S2. HyperSUMO conditions promotes AR SUMOylation and enhances its interaction with Hsp27. Figure S3. SUMO stabilizes AR and reduces its proteasomal degradation. Figure S4. SUMO stimulates basal AR transcriptional activity regardless of the AR-luciferase reporter construct. Figure S5. Concurrent targeting of SUMO-modified and unmodified AR decreases TamR-7 BCa growth in 3D cultures. [file 12964_2020_649_MOESM2_ESM.zip › Fig S3.tiff]

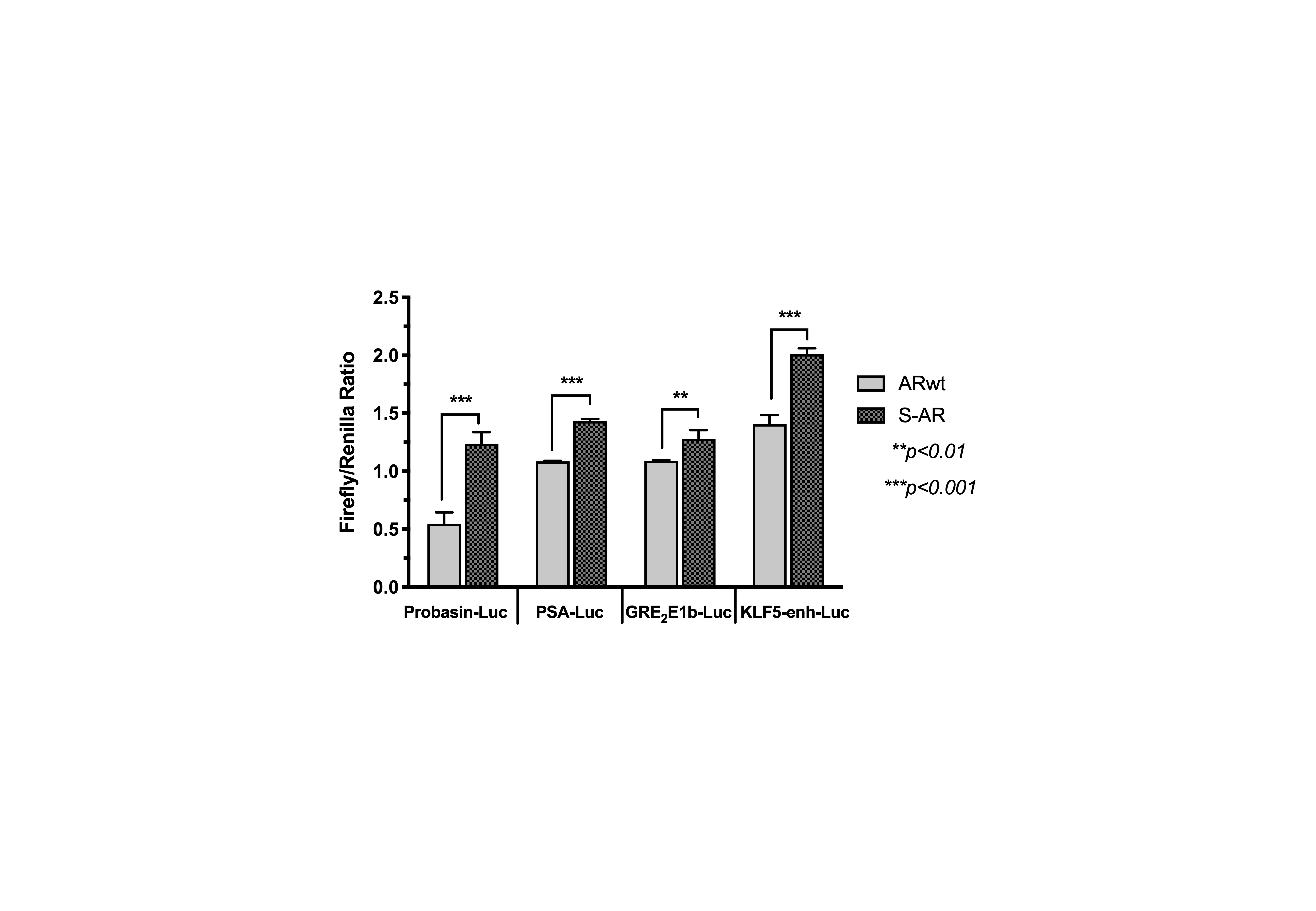

Supplement: Supplementary file 2 — Additional file 1. Supplemental materials and methods: in vitro SUMOylation; RT-PCR; Luciferase reporter assays; PLA, mammosphere studies; scratch assays; transcriptomic data analysis. Table S1. List of primer sequences used for the detection of transcripts. Supplemental figures and figure legends: Figure S1. Elevated levels of SUMO isoforms and HSP27 correlate with high probability of metastasis in ET-treated HR+ BCa patients. Figure S2. HyperSUMO conditions promotes AR SUMOylation and enhances its interaction with Hsp27. Figure S3. SUMO stabilizes AR and reduces its proteasomal degradation. Figure S4. SUMO stimulates basal AR transcriptional activity regardless of the AR-luciferase reporter construct. Figure S5. Concurrent targeting of SUMO-modified and unmodified AR decreases TamR-7 BCa growth in 3D cultures. [file 12964_2020_649_MOESM2_ESM.zip › Fig S4.tiff]

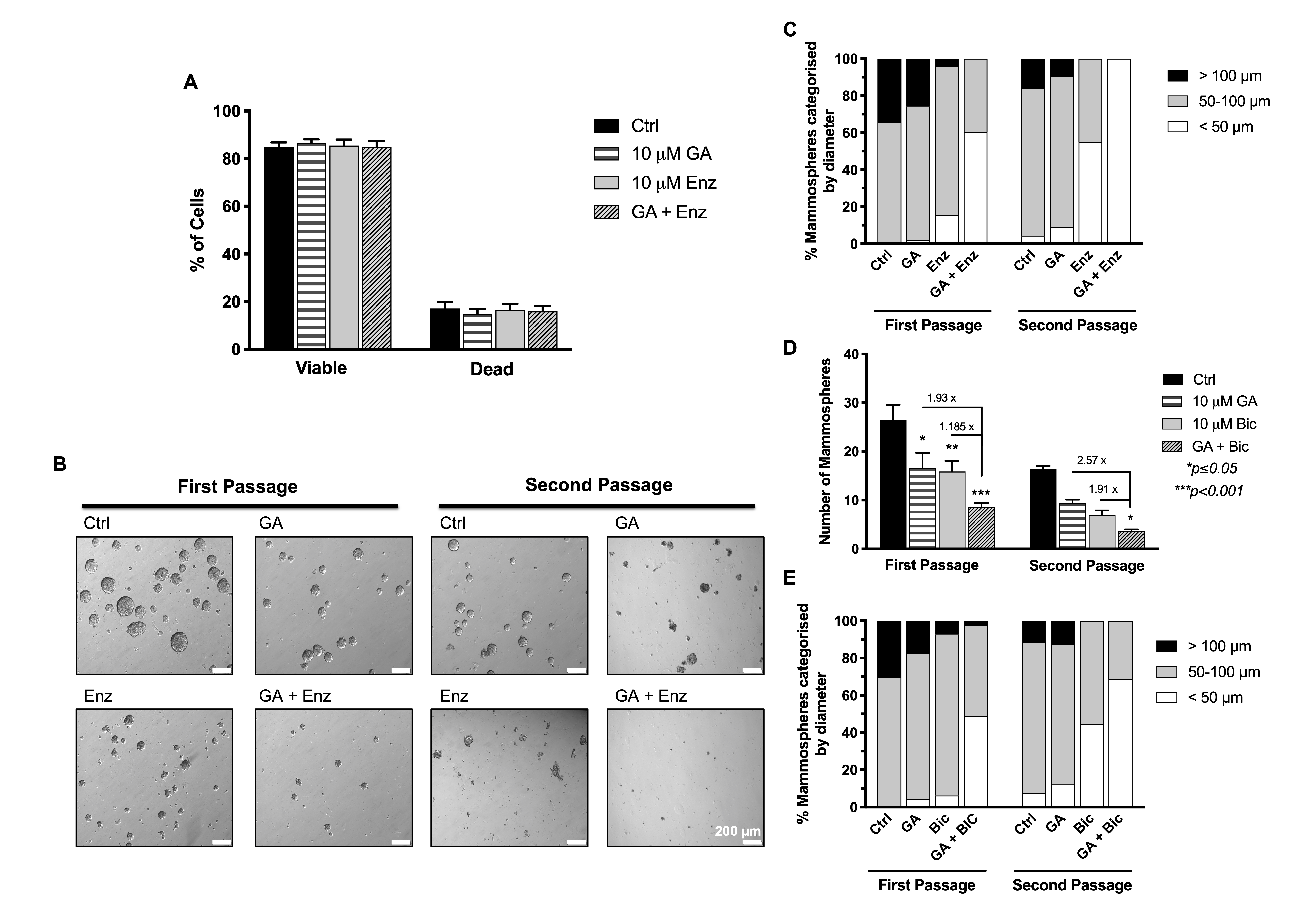

Supplement: Supplementary file 2 — Additional file 1. Supplemental materials and methods: in vitro SUMOylation; RT-PCR; Luciferase reporter assays; PLA, mammosphere studies; scratch assays; transcriptomic data analysis. Table S1. List of primer sequences used for the detection of transcripts. Supplemental figures and figure legends: Figure S1. Elevated levels of SUMO isoforms and HSP27 correlate with high probability of metastasis in ET-treated HR+ BCa patients. Figure S2. HyperSUMO conditions promotes AR SUMOylation and enhances its interaction with Hsp27. Figure S3. SUMO stabilizes AR and reduces its proteasomal degradation. Figure S4. SUMO stimulates basal AR transcriptional activity regardless of the AR-luciferase reporter construct. Figure S5. Concurrent targeting of SUMO-modified and unmodified AR decreases TamR-7 BCa growth in 3D cultures. [file 12964_2020_649_MOESM2_ESM.zip › Fig S5.tiff]
